# Consensus or conflict? Recommender systems as recursive exposure systems in the production of polarization

**DOI:** 10.3389/fsoc.2026.1940431

**Published:** 2026-09-30

**Authors:** Sadiq C. P., Rahul Shukla

**Affiliations:** Department of Social Sciences and Languages, Vellore Institute of Technology, Vellore, Tamil Nadu, India

**Keywords:** algorithmic curation, echo chamber, exposure asymmetry, polarization, recommender systems, recursive systems

## Abstract

Recommender systems are central to debates over whether digital platforms fragment public discourse or sustain exposure to diverse information, yet empirical findings remain heterogeneous. This study examines how filter bubbles, echo chambers, and polarization are conceptualized and operationalized in recommender-system research and integrates evidence across algorithmic, behavioral, relational, and outcome levels. A systematic review of 60 studies published between 2020 and 2025, conducted and reported in accordance with PRISMA, informs the development of the Platform-Mediated Polarization Framework (PMPF). Extending the recursive logic of Reinforcing Spirals Theory to computationally adaptive recommender environments, PMPF conceptualizes recursive exposure production as a process through which behavioral responses to prior exposure may be incorporated into subsequent ranking and personalization. Evidence was more extensive and direct for recommender-mediated structuring and differentiation of informational exposure than for attitudinal or ideological polarization. Evidence for polarization was more heterogeneous, suggesting that such outcomes are contingent on behavioral, relational, informational, and contextual processes. Direct evidence of recursive feedback was limited to a subset of studies, while others examined component processes or addressed feedback conceptually. The evidence therefore does not support a uniform relationship between recommender systems and polarization. Instead, PMPF integrates these patterns within a system-level framework in which exposure conditions may be recursively produced across successive interactions, while their direction and consequences remain open to empirical investigation.

## 1 Introduction

Recommender systems play a controversial role in discussions of digital communication in the present day. On the one hand, these systems have been linked with the polarization of public discourse and, on the other hand, have been associated with diversification of informational environments with empirical support for both positions being found (Flaxman et al., 2016; Dubois and Blank, 2018). Coexistence of apparently conflicting findings has resulted in disagreements concerning the role of algorithmically managed platforms either in broadening of the informational environment or its polarization (Stray, 2022; Nechushtai, 2024). Being increasingly involved in organizing the online content through ranking, personalization, and recommendation, these platforms play an important role in discussions about whether digital platforms help in establishing shared informational spaces or increase ideological polarization (Sunstein, 2017; Bennett and Pfetsch, 2018). Such controversy indicates that the problem under discussion is not merely empirical; it is also conceptual.

Part of the problem is in fragmented research along different analytical dimensions. Some studies focus on algorithmic filtering and ranking, others on individual choice and selection, still others on network interaction and/or attitudinal/ideological outcomes (Del Vicario et al., 2016; Cinelli et al., 2021; Stray, 2022; Nechushtai, 2024). All these perspectives describe important processes, but their fragmentation makes it problematic to explain the interplay of computational, behavioral and relational processes in platform-mediated communication. The theoretical problem is, therefore, in establishing links between these processes while maintaining the analytical distinction between conditions of exposure, relational arrangements, and polarization outcomes.

Such a distinction is especially important in cases of exposure asymmetry, exposure polarization, filter bubbles, echo chambers, and polarization. Exposure asymmetry is in differential visibility or distribution of information, sources or viewpoints in informational environments of users and will be considered here as a characteristic of exposure and not evidence of polarization. Where other research employs the term exposure polarization, in the course of this study it will be treated similarly as differentiation of informational exposure and not as attitudinal/ideological polarization. Filter bubbles are in their turn a particular configuration of exposure where the combination of algorithmic mediation and selection results in narrowing of diversity of information experienced (Pariser, 2011; Bruns, 2019). Echo chambers involve particular relational arrangement defined by homophily, repeated interactions, and social reinforcement (Del Vicario et al., 2016; Cinelli et al., 2021). Polarization is understood as divergence or segregation of users/groups with regard to attitudes, affective orientations, perceptions or ideological stances (Barberá, 2020; Iyengar et al., 2019). Though these phenomena can interact, they are not conceptual substitutes for each other and there is no assumption that they emerge in some particular sequence. This hierarchy will guide analysis throughout the review.

Another difficulty is related to the temporality. Empirical studies tend to focus on bounded periods of platform usage, while recommender environments can change through repetitive interactions. Research on recursive media use and feedback relations of recommendation algorithms indicates that current media use and processes of recommendations can affect the conditions of future interactions (Slater, 2007, 2015; Mansoury et al., 2020; Adomavicius et al., 2022). The information experienced during one period of platform usage can therefore become part of conditions of future interactions and, as such, temporal dimension becomes a factor through which feedback relations can develop. In order to address this problem, the framework to be developed should incorporate changes of exposure conditions across consecutive interactions.

In order to address these problems, the Platform-Mediated Polarization Framework (PMPF) is to be developed in the course of the study. PMPF integrates algorithmic infrastructure, exposure conditions, user practices, network interactions, and polarization outcomes without blurring their analytical distinction. PMPF draws on and does not replace the existing explanations. The theory of selective exposure provides explanation of individual preferences in engaging with information congruent with current attitudes, interests or identities (Garrett, 2009; Stroud, 2011, 2017). Research on algorithmic amplification focuses on the ways ranking and personalization transform the visibility of information (Gillespie, 2014; Bucher, 2018; Stray, 2022), while echo-chamber approaches highlight homophily, repeated interaction, and social reinforcement (Jamieson and Cappella, 2008; Del Vicario et al., 2016; Cinelli et al., 2021). All these approaches cover complementary aspects of platform-mediated communication; PMPF is to incorporate them into one analytical framework for examining their relationships within adaptive algorithmic environments.

Most closely related theory to PMPF is Reinforcing Spirals Theory (RST), which views mutual relations between media selection, media use and individual dispositions as a reciprocal process (Slater, 2007, 2015). However, the proposed framework cannot be seen as a substitute to RST; rather, it is an extension of the latter’s recursive dynamics into computationally adaptive recommender environments. The unique focus of the proposed framework lies in the recursive production of exposure conditions. Behavioral responses to previous exposure—such as viewing, clicking, liking, sharing, or any other kind of interaction signals—can be coded as machine-readable input for future exposure (Mansoury et al., 2020; Adomavicius et al., 2022). Thus, algorithmic updating becomes a part of return relationship, where responses elicited during one exposure environment become the source of the subsequent exposure conditions. In PMPF, the process of algorithmic connection of different interaction states becomes the system level mechanism of connecting behavioral, algorithmic, and relational dimensions.

Importantly, recursive exposure production is analytically separate from polarization. Recursive processes can alter future exposure trajectories without producing attitudinal or ideological polarization. Therefore, polarization can be considered a more distal and conditional phenomenon depending on additional behavioral, relational, informational, and temporal factors. This makes PMPF capable of accounting for various information trajectories without assuming that the process of recommendation always leads to the polarization. At the same time, the distinction between exposure and polarization can facilitate the understanding of heterogeneity of methodologies because different methodologies may address different aspects of these phenomena without making similar assumptions about the downstream effects.

Based on this discussion, PMPF proposes three theoretical propositions:

*Proposition 1*: Exposure – outcome distinction. Changes in exposure conditions are more proximal to recommender processes, while polarization is a more distal outcome dependent on additional behavioral, relational, and temporal factors.

*Proposition 2*: Recursive exposure. When behavioral responses to previous exposure are used for subsequent ranking or personalization, exposure conditions can be replicated, intensified, attenuated, re-directed, or diversified in the course of recurrent recommendation cycles.

*Proposition 3*: Conditional polarization. Recursive exposure processes can lead to polarization through the interplay between algorithmic structure of exposure, selective engagement, network reinforcement, and contextual conditions. The strength and direction of these relationships may depend on platform architecture, content ecology, user characteristics, and temporal conditions.

The study provides the following contributions to knowledge. First, it demarcates the concepts of exposure asymmetry, exposure polarization, filter bubbles, echo chambers, and polarization. Second, it integrates evidence from the algorithms, behavior, relational, and polarization outcomes without reducing the analytical distinctions among these dimensions. Third, it extends the RST’s recursive dynamics into computationally adaptive recommender environments via the concept of recursive exposure production.

Within the framework of PMPF, the study addresses the following research questions:

*RQ1*: What are the definitions of filter bubbles, echo chambers, and polarization in the literature on recommender systems?

*RQ2*: How do these phenomena get operationalized and investigated using various methodological approaches?

*RQ3*: How can evidence from algorithms, behavior, relations, and polarization be integrated into a recursive framework of platform-mediated communication?

Thus, through the answers to these questions, the study reorients the recommender systems literature from a dichotomous question of consensus or conflict into an exploration of conditions of different communicative trajectories.

## 2 Methodology

### 2.1 Review design

The present review utilizes a systematic literature review (SLR). SLRs represent an evidence-based and rigorous methodology to integrate research findings while minimizing bias, improving transparency, and enhancing replicability. In contrast to the narrative literature reviews, SLRs follow predetermined procedures to achieve systematic and replicable synthesis of research literature. The review follows the Preferred Reporting Items for Systematic Reviews and Meta-Analyses (PRISMA 2020) framework (Page et al., 2021), specifying a set of stages including identification, screening, eligibility, and inclusion. Beyond the tool for report formatting, PRISMA serves as an evidential logic, providing systematic, comparable, and transparent inclusion of studies across disciplines. A systematic review is especially appropriate for the current research, as the goal of the study is not only to synthesize research literature but to integrate different findings and build a theoretical framework. This approach will allow the synthesis of studies that reveal different views on recommender systems to identify cross-level mechanisms and feedbacks necessary to understand recursive dynamics behind the theory proposed by this paper.

### 2.2 Search strategy

The structured search strategy was developed to identify contemporary research literature on recommender systems in relation to differentiated information exposure and its communicative consequences. The searches were conducted in Scopus and the Web of Science Core Collection due to their interdisciplinary coverage in the fields of communication, social sciences, information systems, and computational research. The main systematic search included keywords specifying recommender systems and recognized concepts of differentiated, selective, or reinforcing information environments: (“recommender system” OR “algorithmic recommendation” OR “content recommendation” OR “algorithmic personalization”) AND (“filter bubble” OR “echo chamber” OR “selective exposure”)*.

The search strategy was purposefully built around the exposure- and relational-level concepts rather than treating the concept of polarization as equivalent to filter bubbles, echo chambers, or selective exposure. Following the conceptual distinctions used in this review, polarization was treated as an analytically separate and potentially more distant outcome that may arise as a result of interactions among recommender-mediated information exposure, selective engagement, and network processes.

To ensure that the primary search strategy did not systematically underestimate the body of the research explicitly oriented toward polarization, a sensitivity search was conducted in both databases. The keywords related to recommender systems from the primary search strategy were combined with “polarization” OR “polarisation.” The results were compared with the primary evidence base and checked for their substantive relevance to recommender-mediated information exposure and polarization. The sensitivity search revealed a substantial overlap with the literature identified in the primary search strategy, while also identifying additional literature that focused on polarization in the context of recommender systems. Substantively relevant literature identified in the sensitivity search was examined against the analytical categories generated based on the primary literature to see if there is any additional concept, mechanism, or relationship that would change the interpretation of the synthesis. In this way, the sensitivity search served as a robustness check for the conceptual coverage of the primary search strategy rather than as a retrospective enlargement of the initial search scope. The sensitivity analysis did not reveal a substantively distinct mechanism or relationship that would require any modification of the principal analytical categories, however, it provided some additional evidence on some of the processes that were already included in the synthesis.

Thus, the search strategy distinguished between the retrieval and the analytical concepts. Recommender systems, filter bubbles, echo chambers, and selective exposure defined the primary retrieval framework. Polarization was treated as an analytically distinct outcome, while the broader phenomena found in the literature, such as misinformation, radicalization, algorithmic amplification, and platform governance, were analyzed as additional relevant mechanisms, outcomes, or context dimensions, respectively. These phenomena were considered as the analytical dimensions of the retrieved evidence base rather than the independent retrieval or eligibility criteria. The related terminology such as algorithmic curation and information cocoon found in the retrieved literature was documented as the terminology variation but was not used as the additional retrieval terms. This strategy allowed maintaining the transparent and clearly defined boundaries of the systematic evidence base while assessing the sensitivity of the synthesis to alternative polarization terminology. The systematic evidence synthesis was limited to the studies published between 2020 and 2025; the temporal boundary is discussed in the eligibility criteria. The restriction to Scopus and Web of Science is recognized as a limitation related to the database, as relevant research literature available only in other databases may have been missed.

### 2.3 Inclusion and exclusion criteria

The inclusion and exclusion criteria were set up *a priori* to guarantee the consistency in the selection of the studies.

Inclusion criteria:

- Studies examining algorithmic recommender systems or algorithmic personalization.
- Studies discussing information exposure, filter bubbles, echo chambers, selective exposure, or polarization in relation to recommender systems.
- Peer-reviewed journal articles or conference proceedings published in Scopus or Web of Science.
- Studies published between 2020 and 2025.
- Publications in English.

Exclusion criteria:

- Technical studies dealing exclusively with algorithms development, optimization, or performance without examining the impact of recommender systems on information exposure, communication, and other social outcomes.
- Commercial recommendation systems not addressing any aspects of information exposure (for example, product recommendations in online shopping).
- Non-peer-reviewed publications, editorials, opinion pieces, and other document types not fitting the specified eligibility criteria.
- Studies for which the full text is not available.

Studies from other domains such as computer science and other technical disciplines remained eligible if they were related to the information-exposure, communicative, or socio-technical consequences of recommender systems, while the classification discipline of the paper alone was not regarded as an exclusion criterion. In this regard, the review was limited to papers published from 2020 till 2025 in order to conduct the contemporary evidence synthesis in relation to recommender systems in terms of differentiated information exposure and polarization. The year 2020 was set as a scope limitation, but not a technological one. However, earlier works are retained to form the conceptual and theoretical foundation for the review, but do not enter into the scope of systematically synthesized evidence base. Thus, the review is to be regarded as a contemporary evidence synthesis for 2020–2025, not as a historical one in the area of recommender systems and polarization.

### 2.4 Study selection process

- The selection process has followed the four-stage PRISMA procedure.
- Identification: 375 records identified (Scopus = 84; Web of Science = 291).
- Removal of duplicates: 146 duplicates removed → 229 unique records.
- Title and abstract screening: 131 records excluded (98 articles retained).
- Eligibility: 38 articles excluded in the full text review.
- Included: 60 studies in the synthesis (see Figure 1).

**Figure 1:**
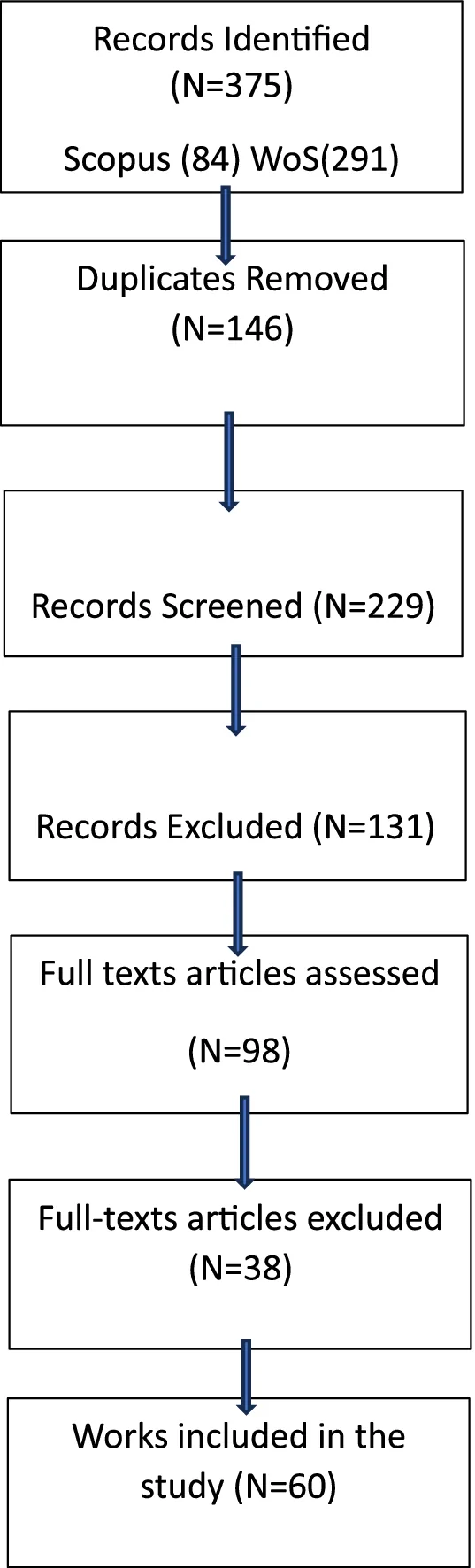
PRISMA flow diagram of the study selection process.

This ensures a transparent and reproducible study selection.

### 2.5 Data extraction

Data extraction protocol was adopted to facilitate consistent comparison of studies of diverse designs. We extracted the following information from each study:

- Author(s) and year of publication.
- Type of the study (experiment, survey, computer simulation, etc.).
- Media platform/environment studied.
- Theoretical framework studied (filter bubbles, echo chambers, polarization).
- Main empirical findings.

This allowed for consistency and methodological heterogeneity in the analysis, thus facilitating both descriptive and more analytical integration.

### 2.6 Analytical strategy

Thematic synthesis methodology was applied to combine evidence from methodologically heterogeneous studies which could not be subject to statistical meta-analysis. The analysis was performed not only as descriptive but also focused on connections between the mechanisms operating on algorithmic, behavioral, and relational levels. The process of thematic synthesis involved three steps. At the first stage, the concept identification was carried out: the recurrent mechanisms in the context of algorithmic exposure, behavioral dynamics, and network interactions were identified. At the second step, the identified mechanisms were grouped according to themes corresponding to the exposure processes, behavioral dynamics, and network interactions. Finally, at the third step, connections and interactions between the identified themes in various studies were explored with special attention to the evidence of feedback between computational, behavioral, and relational mechanisms.

Coding was performed at two levels. On the first level, studies were classified according to the year of publication, media platform used, methodological approach, theoretical construct studied, and main findings. Then, the following mechanisms were coded: algorithmic narrowing, exposure diversity, selective engagement, network reinforcement, misinformation amplification, and polarization-related effects. A mechanism was coded when it was measured, modeled, examined experimentally, or conceptually explained in the context of the theoretical analysis of the paper. When a study investigated several mechanisms, the primary and secondary classification was performed in order to account for cross-level interactions.

Further, the connection between these mechanisms in the evidence base was explored, especially the relationships among algorithmic ranking, exposure, user behavior, network dynamics, and recommendation updating. Recursivity was considered as the property of the relations which required identification of a feedback pathway rather than the co-occurrence of several mechanisms. Specific criteria used for differentiation between direct recursive evidence, partial evidence, conceptual evidence, and non-recursive evidence are presented in section 2.8. Different findings were analyzed in the context of the design, platform, and level of analysis of the study, rather than being treated as inconsistent. It allowed to investigate the circumstances under which similar mechanisms are connected to different exposure or polarization-related effects.

Title/abstract screening, full-text evaluation, and data extraction were first conducted by the first author and later were reviewed by the supervisor, paying special attention to the study eligibility and consistency of data extraction. The first author performed the initial thematic coding while the supervisor reviewed coding decisions, theme borders, and interpretations in order to facilitate consistency in the interpretive synthesis. All discrepancies found at any of the steps were discussed and reconciled. Since initial assessment and supervisory review were performed instead of independent assessment, agreement statistics were not calculated.

### 2.7 Methodological quality assessment

The methodological quality was assessed for all included studies using a specially designed design-sensistive assessment rubric. Six domains were assessed: (1) appropriateness of research design; (2) adequacy of the sample/dataset/other empirical base relative to the study design; (3) transparency and rigor of data collection/experimental procedure/model construction; (4) appropriateness and transparency of analysis; (5) conceptual and operational clarity; and (6) consistency between the evidence and conclusions. The criteria were interpreted according to the study design: e.g. computational models, simulations and audits were assessed in terms of the adequacy of the dataset, model assumptions, validation procedure, data coverage, etc., but not conventional participant representativeness.

Each domain was rated as 0 (weak), 1 (adequate) or 2 (strong), resulting in a possible score range of 0–12. Scores of 10–12, 7–9, and 0–6 were categorized as higher, moderate, and lower methodological quality, respectively. These categories were review-specific indicators but not externally validated and they were not applied as exclusion criteria. Out of 60 included studies, 38 received the rating of higher quality, 20—of moderate quality, and two—of lower quality with the mean score of 9.8/12. Individual study ratings and classifications are provided in Supplementary Table S1.

The initial quality assessment of all included studies was conducted by the first author, and the supervisor subsequently reviewed the assessments. Disagreements found during this process were reconciled through discussions. The methodological quality was not used to exclude studies or statistically weight findings; instead, methodological limitations identified facilitated the interpretation of evidentiary strength during the synthesis. The methodological quality was assessed separately from the directness of evidence for recursive dynamics and polarization-related outcomes. A methodologically strong study did not necessarily provide recursive feedback or evidence on polarization-related outcomes; these dimensions were, therefore, evaluated separately during the analysis.

### 2.8 Assessment of recursive evidence

In order to distinguish the co-occurrence of several related mechanisms from the evidence on an actual feedback relationship, recursive evidence was assessed using a relationship-based criterion. A study was classified as direct recursive evidence only if the return pathway through which an output or behavioral response from one interaction cycle feeds into a subsequent recommendation, ranking, exposure, or network state was observed, experimentally traced, or computed in a model. Thus, the co-occurrence of several related processes in a study does not necessarily imply the evidence on recursion without an identifiable return pathway.

The studies were classified into four groups of evidentiary classes: (1) Direct recursive evidence, if the return pathway between successive interaction states is empirically observed, experimentally traced, or computed in a model; (2) Partial/component evidence, if several components of the recursive process are examined but the return pathway is not established; (3) Conceptual/theoretical recursivity, if the feedback dynamics are explicitly theorized or discussed without direct empirical evidence; and (4) No recursive evidence, if the feedback pathway and/or the theoretical formulation of recursivity are not identified.

All included studies were classified according to these criteria, paying attention to the design, temporal structure, the treatment of the user behavior/interaction, presence or absence of the identifiable return pathway, and the extent to which the feedback dynamics were empirically examined or theoretically described. This classification allowed to distinguish the evidence on recursive dynamics from the evidence of the separate components of it which were further theoretically combined in the PMPF.

## 3 Analysis of the included studies

### 3.1 Characteristics and evidentiary features of the corpus

The 60 included studies comprise a heterogeneous evidence base in terms of methods used. Experiments were the most frequent method type (*n* = 23), followed by modeling and simulation (*n* = 14), design of algorithms or systems (*n* = 8), measurement/empirical modeling (*n* = 7), audit (*n* = 3), reviews/bibliometrics (*n* = 3), and qualitative studies (*n* = 2). This heterogeneity is relevant for interpretation since each method type provides different types of evidence: modeling and simulation studies show plausibility of mechanisms under the specific assumptions made, while experiments analyze the bounded user response to particular changes. Audit studies and platform studies provide a closer look at recommender outputs and exposure design, while qualitative studies shed light on the way users interpret algorithms’ mediations (Aridor, 2020; Donkers, 2021; Han, 2022; Knudsen, 2023; Jeng, 2024; Bojic et al., 2025). The corpus should be considered, thus, not as a set of equivalent studies of one causality but as a heterogeneous evidence base. The main features of the corpus and their evidentiary implications are shown in Table 1, while the complete mapping of individual studies is presented in Supplementary Table S2.

**Table 1:** Evidence profile of the included studies and implications for the PMPF.

Evidence dimension	Corpus pattern	Evidentiary contribution	Implication for PMPF
Study design	Experiments (23); modeling/simulation (14); system/algorithm design (8); empirical modeling (7); audits (3); reviews/bibliometric (3); qualitative (2).	Different designs capture mechanism plausibility, user responses, observable recommender outcomes, and interpretive experience.	PMPF rests on heterogeneous forms of evidence rather than a single type of causal test.
Exposure-level evidence	Prominent across news, general recommender, and platform studies.	Shows changes in visibility, diversity, concentration, ranking, and exposure opportunities.	Strongest support concerns structuring of exposure conditions rather than polarization itself.
Behavioral and relational evidence	Experiments examine selective engagement; network studies examine homophily, clustering, and reinforcement.	Shows that exposure effects depend on user uptake and network interaction.	Supports behavioral and relational components while preserving their distinction from algorithmic exposure.
Polarization evidence	Smaller and methodologically heterogeneous cluster; outcomes vary across exposure, ideological, affective, and perceived forms.	Evidence is conditional and more distal than exposure-level effects.	Polarization is treated as a contingent outcome, not an automatic effect of recommendation.
Recursive evidence	Direct 14 (23.3%); partial/component 11 (18.3%); conceptual/theoretical 14 (23.3%); none 21 (35.0%).	Only a subset directly demonstrates a return pathway across successive interaction states.	The recursive architecture is partly directly demonstrated and partly theoretically integrated from component evidence.
Methodological quality	Higher 38; moderate 20; lower 2; mean 9.8/12.	Most studies provide moderate-to-higher methodological support, but quality and evidentiary directness are distinct.	A strong study may still provide no direct evidence of recursive feedback.
Overall evidence pattern	Evidence is stronger for exposure structuring and constituent mechanisms than for long-term polarization.	Directness decreases as analysis moves from proximal exposure processes to distal outcomes.	PMPF is an evidence-informed integrative framework, not a fully tested causal model.

Recursive-evidence categories follow Section 2.8; methodological-quality categories follow Section 2.7. Methodological quality and evidentiary directness are treated as analytically distinct. The complete study-level evidence map is reported in Supplementary Table S2.

Across these methodological approaches, the evidence is most comprehensive and comparatively direct in relation to recommender-mediated exposure conditions. In relation to news recommender studies, there is extensive research on changes in visibility and exposure through topic diversity, source diversity, viewpoint balance and other measures, rather than on direct measurement of opinion transformation (Joris, 2020; Vrijenhoek, 2020, 2021; Michiels, 2023; Treuillier, 2024; Wang, 2025). General recommender system studies focus on trade-offs between relevance, diversity, fairness, novelty and serendipity (Sun, 2020; Zhao, 2021; Wang, 2022; Gao, 2024; Kim, 2025; Xi, 2025). Social-network studies include the relational perspective on homophily, community structure, social recommendation and reinforcement (Grossetti, 2021; Morales, 2021; Tommasel, 2021; Duskin, 2024; Pathak, 2024a,b). Collectively, these studies show that recommender systems have the ability to structure the information environment of users. However, evidence that differences in exposure translate into attitudinal or ideological polarization is less consistent.

This difference is also reflected at different levels of analysis. The studies oriented toward changes in exposure capture system-level evidence, such as changes in ranking, diversity of recommendation, information concentration and exposure opportunities. Experimental and user-oriented studies extend the evidence base to behavioral responses, including selective exposure, engagement, uptake and response to diverse or congruent recommendations (Knudsen, 2023; Jeng, 2024; Sergas, 2024; Sonoda, 2024; Jiang, 2025). The network-oriented studies examine the way how exposure and engagement may interact with homophily, clustering and social reinforcement (Grossetti, 2021; Tommasel, 2021; Duskin, 2024; Pathak, 2024a,b). Studies examining polarization, misinformation and radicalization cover evidence on outcomes that are more distal analytically to the recommender system output, but their findings are relatively heterogeneous across methodology and platforms (Donkers, 2021; Ludwig, 2023; Nechushtai, 2024; Shin, 2024; Schellingerhout, 2025). Thus, the evidentiary structure of the corpus does not allow treating exposure asymmetry, network reinforcement and polarization as equivalent outcomes. Instead, they are distinct analytically and the evidence available for each of these processes is distinct as well.

The thematic structure of the corpus is similarly consistent with recurring interest in filter bubbles, information cocoons, exposure diversity and echo-chamber effects, although these categories are not mutually exclusive. Selective exposure is mentioned less often as an explicit coding category but reappears in studies covering the interaction between recommender-structured exposure and users’ engagement. Studies focusing specifically on polarization, misinformation and radicalization are a more heterogeneous part of the evidence base. The reviews and mapping studies illustrate similar attention to exposure differentiation, information cocoons and conditions under which informational narrowing may develop (Areeb et al., 2023; Yan, 2025; Ahmmad, 2025). Overall, the thematic composition of evidence provides more evidence for conditions of informational narrowing, homophily and reinforcement than for any consistently established relationship between recommender-system exposure and ideological polarization.

The evaluation of the evidence for recursive process further illustrates the relationship between systematic evidence and the PMPF. Out of 60 studies included, 14 (23.3%) studies provided direct recursive evidence, in which return pathway between successive interaction states was empirically observed, experimentally traced or computationally modeled. Eleven studies (18.3%) provided partial/component evidence, covering several elements of recursive process but not establishing the return pathway, while 14 (23.3%) examined recursivity at conceptual/theoretical level. The remaining 21 studies (35.0%) did not provide recursive evidence in accordance with the criteria covered in Section 2.8. Direct demonstrations of recursive relations thus remain concentrated in a limited subset of the corpus, not distributed evenly throughout the literature. Study-level classification is reported in Supplementary Table S2.

In the quality evaluation, methodology and evidentiary directness were considered separately. Quality assessment classified 38 studies as high quality, 20 as moderate quality and two as low quality. The average score on methodology quality out of 12 was 9.8. Such classification reflects the strength of methodology in relation to the study design, but does not reflect whether a study provides the evidence for recursive interaction. A high-quality experiment, for example, can give a robust demonstration of behavioral response without showing that response affects recommendation conditions afterwards. Modeling study can explicitly incorporate recursive loop, while giving the evidence of mechanism plausibility, rather than of actual user behavior. Thus, maintaining this distinction allows to keep methodology quality from being conflated with direct evidence of the whole PMPF architecture. The study-level quality assessments are reported in Supplementary Table S1.

Thus, the corpus of evidence is asymmetrical with respect to the framework proposed. It includes extensive and direct evidence for recommender systems structuring exposure, and for mechanisms that mediate or reinforce it. Less direct evidence is available for the complete recursive relations and polarization-related outcomes than for the exposure-level processes. The PMPF thus cannot be presented as causal model that is directly supported by the evidence from all 60 studies. Instead, it integrates the direct evidence of recursive interactions, components of the model supported by the evidence and feedback mechanisms theoretically identified, to give a systems-level account where different relationships vary in evidentiary directness. This hierarchy of evidentiary base serves as the foundation for the mechanism-specific synthesis performed in the subsequent sections.

### 3.2 Mechanism 1: filter bubbles as exposure concentration

One of the dominant threads of the corpus looks at filter bubbles in terms of shifts in the diversity and composition of recommended exposure. Rather than relying on the concept as a metaphorical representation of informational encapsulation, the literature investigates the concrete properties of information environments, including diversity of topics and viewpoints, distribution of exposure, and similar variables. For instance, Lunardi (2020) and Michiels (2023) devise or employ filter-bubble measures, whereas Vrijenhoek (2020, 2021) explores variations in viewpoint or topic diversity in news recommender environments. Joris (2020) offers a broader mapping of measures and research gaps, whereas Reid (2024) and Poechhacker (2024) deal with exposure diversity and homogenization from the perspective of modeling or system design. Altogether, these works draw attention to ways in which information narrowing may be investigated, conceptualized, and compared across recommender environments. In this regard, exposure concentration is understood as a shift in the diversity and distribution of information exposure produced through recommendation without the necessity of blocking access to any other information.

In this literature, the evidence seems to be more consistent with differentiated or narrowed exposure rather than total informational enclosure. For example, Vrijenhoek (2020, 2021), Treuillier (2024), and Wang (2025) document or investigate variations in viewpoint, topic, or exposure diversity. Thus, the filter-bubble issue should be seen not as a binary phenomenon but rather as a gradient, where alternative information does not have to be completely absent for recommendation to produce differences in visibility and likelihood of being discovered. Exposure concentration therefore implies changes in distribution of informational visibility rather than complete information hermeticity.

As far as recursive processes in this mechanism, their nature is somewhat heterogeneous. Out of all studies of filter bubbles and exposure diversity, Wang (2022), Gao (2024), Wang (2025), and Zheng (2025) are considered to provide direct evidence of recursivity. On the other hand, Aridor (2020), Lunardi (2020), Michiels (2023), and Ren (2025) give partial or component evidence, whereas Joris (2020), Vrijenhoek (2020, 2021), Reid (2024), Poechhacker (2024), and Xi (2025) discuss recursivity mainly theoretically or conceptually. This difference matters analytically since personalization, preference learning, or repeated recommendation cannot be taken as evidence of recursivity on its own. To be direct evidence of recursion, one has to show the existence of a feedback loop that impacts recommendation and exposure based on prior experience.

Another aspect is distinguishing changes in the recommendation output and changes in actual exposure or consumption. Intervention-oriented literature addresses the question of whether diversification or alternative designs of recommendation could lead to reduced homogeneity and increased exposure (Sun, 2020; Wang, 2022; Gao, 2022, 2024; Treuillier, 2024; Xu, 2024; Han, 2025; Ren, 2025; Xi, 2025; Zheng, 2025). Altogether, these studies investigate the effect of recommendation designs on specific aspects of exposure diversity, but changes in the recommendation outputs do not imply changes in users’ exposure directly. The evidence from behavioral studies reinforces this point. Knudsen (2023) shows that selective-exposure effects are contingent on prior ideology and context of recommendation. Sonoda (2022, 2024) deals with diversity-related interventions on relational and behavioral level. Jiang (2024, 2025) links algorithmic curation and recommendation designs to selective information consumption, exposure-diversity or filter-bubble results, and attitude extremity. Altogether, this evidence warns against the equating of the increase in diversity of recommendation output to the increase in actual information consumption diversity.

In summary, the evidence in this corpus seems to be sufficient to interpret filter bubbles as a socio-technical process of exposure concentration rather than a guaranteed condition of information enclosure. Recommendation designs could create certain informational opportunities, but actual exposure of users depends on engagement and context of recommendation. Therefore, the evidence about changes in exposure composition and diversity is more numerous and direct than the evidence about persistence or further consequences of those changes. The evidence about persistence of exposure concentration over time, its recursive reproduction, and contribution to polarization is less uniform. Inside PMPF, this evidentiary difference allows one to treat exposure concentration as a proximal process of recommender-mediated filter bubble formation.

### 3.3 Mechanism 2: echo chambers as networked reinforcement

Unlike the literature on filter bubbles, which mostly focuses on composition and concentration of the recommender-mediated exposure, echo chambers literature emphasizes a different relational mechanism based on network structure, homophily, clustering, and network reinforcement. Research conducted in the context of social media and social networks analyzes the functioning of the recommendation process within the framework of patterns of interaction between users and communities. Donkers (2021), Grossetti (2021), Tommasel (2021), De Biasio (2023), Du (2023), Duskin (2024), Sonoda (2024), Alam (2025), and Yu (2025) and other scholars investigate network clustering, relational recommendation or echo chamber effect. Therefore, the echo chambers are considered here as relational configurations of information circulation and exposure mediated by the patterns of connection and interaction between users.

This relational approach implies making distinctions between content recommendation, social recommendation and homophily. Despite the fact that these processes can interact, they are sources of different types of exposure structure. Grossetti (2021) investigates how network ranking promotes homophily and how diversification can prevent this tendency, while Tommasel (2021) discusses diversification as part of social recommendation. De Biasio (2023) also examines network clustering through system design, while Morales (2021) and Duskin (2024) analyze the interaction between recommender environment and clustering or informational steering through the analysis of platform. Overall, these studies demonstrate how network structure can act both as a condition for recommendation and as a factor potentially affected by recommendation processes. It prevents the automatic attribution of pre-existing relational similarity to algorithmic recommendation, while allowing for its interaction with the latter.

The evidence that such relational processes are recursive is quite limited and uneven. Donkers (2021), Morales (2021) and Duskin (2024) are considered as providing direct recursive evidence, while Grossetti (2021), Tommasel (2021), De Biasio (2023), Du (2023) and Yu (2025) as providing partial or component evidence related to networked reinforcement. Meanwhile, Sonoda (2024) and Alam (2025) investigate relational or clustering processes without demonstrating the recursive return path. Therefore, homophily, clustering and repeated interaction cannot be considered as evidence of recursion. The evidence provided by the corpus includes only some of the investigated relational studies in terms of their direct recursive interaction, while the others prove component-level relations.

Moreover, this evidence shows that such configurations are design-sensitive rather than technological ones. Grossetti (2021), Tommasel (2021), De Biasio (2023), Sonoda (2024) and Yu (2025) investigate diversification or recommendation strategy in relation to homophily, clustering or relational exposure. Thus, these studies analyze the ways recommendation design can change some exposure or relational conditions, but they do not confirm that the diversification leads to durable restructuring of social networks. This distinguishes diversification of recommendation from the change of relational structures, through which the information is shared.

In general, the evidence allows considering the echo chambers as networked reinforcement rather than another form of exposure concentration. While filter bubble processes focus on information visibility and diversity, echo chamber ones are concentrated on the organization of relational circulation and interactions. The two mechanisms can intersect if recommendation functions in homophilous or clustered networks, but one does not necessarily result in the other. In the PMPF, networked reinforcement is considered as a relational mechanism of recommender-mediated exposure reproduction/reconfiguration within the framework of successive interactions; however, whether this dynamic results in recursive relations or polarization depends on the platform architecture, network structure and patterns of user interaction.

### 3.4 Linking exposure and engagement: exposure diversity and selective exposure

Selective exposure holds an analytically significant but relatively implicit place in the corpus. Knudsen (2023) provides an explicit analysis of the conditional nature of selective-exposure effects based on prior ideology and conditions of use. All other studies focus explicitly on the exposure element of the relationship by referring to viewpoint, topic, or exposure diversity but not selective exposure (Vrijenhoek, 2020, 2021; Jeng, 2024; Wang, 2025). Accordingly, evidence is insufficient to treat selective exposure as a mechanism analyzed throughout the corpus. It rather highlights the analytically significant distinction between the opportunities provided through recommendations and users’ reactions to these opportunities.

As discussed in Section 3.2, exposure diversity describes the informational opportunities generated through recommendation systems, including viewpoint or topic diversity. In this context, its role is not to reaffirm the impact of recommendation systems on the narrowing or broadening of exposure but to distinguish information exposed from the subsequent engagement of users. That is, realized exposure refers to the opportunities offered through recommendation which users take up, whereas selective engagement deals with the way users react to the offered informational opportunities. The question here is not related to exposure diversity as such but to the relationships between recommender-structured opportunities and user engagement. Evidence oriented toward users’ behavior emphasizes the significance of this difference. For example, Knudsen (2023) demonstrates that selective-exposure effects are conditional on prior ideology and conditions of use, and Sonoda (2022, 2024) analyses interventions aimed at diversity in relational and behavioral terms. Altogether, these studies suggest avoiding the assumption that there is a straightforward relationship between recommender-structured informational opportunities and user behavior. It is also inappropriate to consider selective engagement as a proof of recursion since relevant behavioral studies do not belong to the group of the studies that offer direct recursive evidence.

All things considered, the evidence supports the distinction between exposure diversity as a property of the informational opportunities and selective exposure as a user behavior which shapes the actual informational opportunities. Such a distinction allows avoiding attribution of realized exposure to recommender processes or user selection separately. In the PMPF, it establishes a link between recommender-structured informational opportunities and user engagement without conflating exposure, behavior, recursion, and polarization.

### 3.5 Outcome cluster 1: polarization as conditional, heterogeneous, and method-sensitive

Studies investigating the topic of polarization show no consistent pattern of a uniform relationship between recommender systems and outcomes related to polarization. Depending on the methodology used, platform context, and specific outcome of interest, findings can vary significantly. Evidence related to polarization comes from modeling and simulation, experimental research, audits, empirical measurements, and literature review, including polarization trajectories, clustering, and polarization-related exposure (Donkers, 2021; Morales, 2021; Han, 2022; Ludwig, 2023; Nechushtai, 2024; Shin, 2024; Bojic et al., 2025; Ludwig, 2025). Taken as a whole, the findings suggest a conditional and heterogeneous nature of polarization dynamics in recommender systems, rather than an inevitable transition from personalization to polarization. The full study-level mapping is presented in Supplementary Table S2. There is one key difference in the evidence on polarization in recommender systems—the difference between polarization-related exposure conditions and actual polarization outcomes. Some papers analyze viewpoint diversity, information distribution, clustering, or polarization-related exposure without showing corresponding attitudinal or ideological polarization (Stray, 2022; Ludwig, 2023; Zhang, 2023; Jeng, 2024; Nechushtai, 2024; Sergas, 2024). Thus, the corpus shows more evidence of polarization-related exposure conditions than evidence of attitudinal or ideological polarization. While the former is potentially relevant for polarization process, it does not mean that there is a polarization per se.

Another factor explaining the variety of results is the methodology used. In the case of modeling and simulations, researchers are able to find out under what conditions the dynamics of polarization can occur. Donkers (2021), for instance, studies echo-chamber dynamics and polarization trajectories, while other modeling studies investigate the effects of different recommender configurations on polarization (El-Moutaouakkil, 2022; Han, 2022; Sergas, 2024; Bojic et al., 2025). Experimental research gives evidence under bounded conditions. For instance, Ludwig (2025) finds bounded and context-dependent polarization associated with ideology-based recommendation, while other experimental papers reveal polarization-related change without showing the uniform transition to ideological polarization (Ludwig, 2023; Zhang, 2023; Lechiakh, 2024). Audits show evidence of clustering, informational steering, or potentially homogeneous exposure, but not attitudinal polarization (Morales, 2021; Nechushtai, 2024; Shin, 2024). Therefore, seemingly contradictory findings actually reflect different levels and conditions of polarization-related dynamics.

In general, the corpus demonstrates that polarization is a conditional and relatively distal outcome, rather than an inherent feature of recommender systems. The relationships depend on platform context, methodology used, and specific recommender configuration. Consequently, within the PMPF, the mechanisms described above are seen as conditions for polarization, not evidence of it.

### 3.6 Outcome cluster 2: misinformation and radicalization as context-dependent risks

The next subset of the corpus consists of papers addressing the topic of recommender systems and their relation to misinformation, conspiracy content, and pathways toward radicalization. This subset includes Valdez (2020); Pathak (2023); Duskin (2024); Pathak (2024a,b); Shin (2024); Schellingerhout (2025); Zhang (2025); and Pathak (2025). In all of these studies, recommendation is analyzed in relation to amplifying, steering or mitigating potentially harmful information. Hence, this subset extends the domain of outcomes studied by the corpus beyond exposure differences to include misinformation and harmful-content pathways.

These studies do not provide any evidence supporting an assumption of homogeneity of outcomes such as misinformation or radicalization resulting from the usage of recommender systems. For example, Valdez (2020) analyzes misinformation amplification and mitigation, while taking into account an interaction between human and algorithmic factors. Further evidence of harmful exposure pathways comes from audits and experiments. In particular, Shin (2024) detects recommendation-related patterns of misinformation amplification and informational steering via the platform audit. Schellingerhout (2025) examines personalization via experiments related to either homogeneous or conspiratorial exposure. There are other studies examining the amplification or mitigation of misinformation and radicalization-related content in social networks, news feeds, and general recommender systems (Pathak, 2023, 2024a,b, 2025; Zhang, 2025). Overall, these studies indicate that recommendation-related outcomes depend on an informational context rather than on the recommendation architecture per se.

This cluster introduces an important evidentiary distinction. Presence of the evidence for potential misinformation-related exposure or amplification cannot be automatically considered as the evidence of users’ acceptance of the misinformation or their becoming radicalized. The corpus provides much stronger evidence of the recommendation-related pathways via which misinformation, conspiratorial or other potentially harmful content can be amplified, steered, or mitigated than of the causal link between recommender systems and radicalization. Hence, exposure to the harmful information, its acceptance, and following radicalization should be considered as distinct outcomes analytically.

Overall, this corpus demonstrates that misinformation and radicalization should be considered as context-dependent risks rather than inherent outcomes of recommendation. In terms of PMPF, informational context becomes a conditional factor for this subset of the corpus: the same recommendation processes may result in different risks in relation to the different informational contexts. This interpretation does not imply a necessity of progression from the exposure to the information acceptance or radicalization.

### 3.7 Moderators and sources of divergence

One of the key drawbacks of simple reviews is the implicit assumption that the field has arrived at one definite answer. This is definitely not the case when it comes to the 60 articles in the corpus. Instead, what it demonstrates is that there are certain systematic drivers of divergence. The first one is method. Generally, the level of polarization potential is higher in simulation and modeling studies due to the fact that the latter concentrate on mechanisms and are able to produce causal trajectories within controlled assumptions (Donkers, 2021; El-Moutaouakkil, 2022; Han, 2022; Lechiakh, 2024; Bojic et al., 2025). Effects tend to be conditional as demonstrated by experimental studies, especially those with real users: Knudsen (2023), Ludwig (2023), Jeng (2024), Sergas (2024), Sonoda (2024), Ludwig (2025), and Jiang (2025). Those papers that stand in-between extremes of these methods are audit studies and platform studies, which reveal structural sorting and clustering, but not necessarily ideological change in the long run (Morales, 2021; Nechushtai, 2024; Shin, 2024; Duskin, 2024). There is no arbitrary disagreement in the literature, but there is a certain degree of structure in how various methods are able to observe. The recursive feedback dynamics are observable in simulation studies, evidence can be found in partial manner in audit, experimental and observational studies through exposure asymmetry, in engagement-based reinforcement and network clustering. This means that while there are certain benefits that arise from the use of one method or another, they are not used directly, but rather observed indirectly, with the complete temporal evolution of such a phenomenon usually not being measured.

The second driver of divergence is the context of the platform. News recommender studies tend to look into whether being exposed to news makes people close-minded or makes their opinion rigid, and thus often make more claims about the former rather than the latter (Lunardi, 2020; Vrijenhoek, 2021; Knudsen, 2023; Jeng, 2024; Wang, 2025). Homophily (Morales, 2021; Tommasel, 2021; Grossetti, 2021; Pathak, 2024a,b) or clustering, and community reinforcement (Morales, 2021; Tommasel, 2021; Grossetti, 2021; Pathak, 2024a,b) are the more common topic of Twitter/X and social-network research. There is another layer of complexity brought by the specialized platforms. Lieto (2023) demonstrates that recommendation in non-political domains can be diverse and inclusive. The discussion of algorithmic news recommender in Eastern Europe goes beyond limiting of exposure and also includes freedom of information normatively, as demonstrated by Makhortykh (2023). Bubble-bursting concept gets elaborated upon in the domain of conversational recommender system, where the mitigation strategy may depend on the type of the platform, as shown by Zheng (2025).

Thirdly, time horizon matters greatly. Much of the research concentrates on short interaction windows or modeled sequences, rather than prolonged sequences. This is important because many of the serious harms that have been documented in the literature are cumulative (polarization, radicalization, epistemic vulnerability). Differences in the results may be caused by the fact that the studied time horizons were different in the case of the output changes (immediate), user behavior (short-term), or pathway stability (long-term) (Donkers, 2021; Han, 2022; Ludwig, 2023; Shin, 2024; Pathak, 2025).

The fourth driver is intervention type. Design studies seem to show that diversified or ranked output, with emphasis on fairness, may relatively effectively reduce the narrowing effect (Wang, 2022; Gao, 2024; Treuillier, 2024; Yu, 2025; Zheng, 2025), whereas diagnostic and user-behavior studies show that such an intervention does not necessarily lead to changes in the user’s interactions with the diverse output (Knudsen, 2023; Du, 2023; Sergas, 2024; Sonoda, 2024; Kim, 2025). Zhou (2024) further examines a user-facing embodied AI learning intervention designed to address filter-bubble formation by increasing users’ understanding of diversity in recommendation systems. This is one of the major tensions that exist in the literature. It does not deny the importance of intervention studies, but indicates that the field has more evidence about changes in the recommendations themselves rather than changes in dispositions of users or their long-term consequences. Highlighting these differences helps to understand the reasons why one should neither be alarmed nor reduce the corpus to one of these extremes. The literature is not unified, but not chaotic either: disagreement is patterned and open to interpretation, which is a sign of maturity of the field.

### 3.8 Intervention and redesign

One particular aspect of the corpus looks at intentional design interventions that are aimed at modifying recommendation and exposure related outcomes. In each of the three categories of experiments, modeling, and system design, the approaches to addressing diversity, serendipity, homogeneity, and the balance between diversification and relevance are intervention oriented. Some work such as that by Sun (2020), Wang (2022), Gao (2024), Treuillier (2024), and Xu (2024) focuses on designing to increase diversity in recommendations, minimize homogeneity, or influence exposure-related outcomes; other work such as Han (2025), Kim (2025), Ren (2025), Xi (2025), and Zheng (2025) is concerned with related designs aimed at creating diversity or serendipity. Research on “relational aspects” of recommendation is also intervention-oriented. Grossetti (2021) and Tommasel (2021) explore the approaches of homophily/homogeneity, and De Biasio (2023), Sonoda (2022, 2024), and Yu (2025) explore the approaches of diversity/serendipity in relational recommender settings. These studies represent the first-time interventions-oriented research has been considered in relation to processes of homophily and clustering beyond the composition of recommended content.

In this evidence, outcomes of redesign are considered primarily at the system, exposure or relational level. The corpus reveals that deliberate design changes can lead to different exposures of recommendations and hence to different recommendation diversities and homogeneities than to corresponding reductions of polarization. There are also differences in the goals of intervention approaches; some boast diversification, and others explore diversity or serendipity with respect to recommendation homogeneity.

Overall, the results reveal that recommender design is a place where deliberate changes can be made, and that there is a clear separation between changes to recommender mediated conditions and changes in attitudinal outcomes downstream. In the PMPF, redesign is therefore understood as an action, not as evidence for depolarization, applied to certain conditions of recommendation. These interventions have wider normatively and governance-related implications, which are considered in the discussion.

### 3.9 Integrative synthesis and answer to the central research question

All the 60 studies indicate an asymmetric and heterogeneous pattern of evidence. Studies that are most extensive and relatively direct are those that investigate structuring of recommender-mediated exposure; behavioral and relational studies investigate intersections of exposure conditions and user engagement and network structure. Evidence is less consistent when it comes to polarization and other downstream effects across methodological designs, platform contexts, and informational contexts (Aridor, 2020; Grossetti, 2021; Knudsen, 2023; Jeng, 2024; Shin, 2024; Ludwig, 2025). There is thus a distinction between recommender mediated exposure situations and their downstream effects, and an evidential directness increases at the recommender mediated exposure situation.

This is a generic way to respond to the main question of how recommender systems can create consensus or exacerbate conflict. Consensus is not sufficiently studied in the corpus and, thus, its relative scarcity should not be viewed as a downfall of recommender systems’ ability to support consensus. Recommendation has shown to have a clearer effect on visibility and dissemination of information and can intersect with user engagement processes as well as with relational processes like homophily and clustering (Knudsen, 2023; Tommasel, 2021; Grossetti, 2021; Pathak, 2024a,b). Such interactions can either reinforce or reconfigure prevailing information and relationship patterns but may not necessarily lead toward consensus or conflict.

While the recursive relationships are directly and unambiguously demonstrated in a subset of the corpus, recommender mediated exposure should not be presumed to be a self-reinforcing recursive relationship. If behavioral responses to previous exposures are included in subsequent exposures, then later exposures can replicate, amplify, weaken or redirect previous exposures. In the PMPF, these return loops can be used as a way to think about information pathways that are different, but not assumed to be the polarization of recursive pathways.

That a recommender system does not inevitably create conflict is therefore not the conclusion that should be drawn from the review, but rather that the system creates “informational opportunities” that have outcomes that are “conditional.” Polarization and other outcomes related to conflict are more distal in relation to recommender outputs and exposure conditions, and rely on effects of interactions between recommender design, user behavior, relational structure, and informational context. The PMPF reformulates the central question from consensus versus conflict, to when exposure to a recommender system can be a source of alternative social and informational paths.

## 4 Discussion

Based on the review, it appears that the relation between recommender systems and polarization is not a consistent one but rather dependent on certain conditions. In particular, the direct evidence in this context is found in the reviewed articles concerning exposure conditions mediated by recommender systems. The evidence of recursive relations and polarization as a consequence is somewhat inconsistent and indirect, and evidence of behavior and relational aspects of the relation are even less present and consistent. It suggests that the review demonstrates a hierarchical structure, with the support becoming increasingly weaker moving from exposure conditions to recursive relations and attitudinal outcomes.

This evidentiary hierarchy is the starting point for the Platform-Mediated Polarization Framework (PMPF). PMPF is not supposed to be an alternative to already established theories of media selection, reinforcement, and platform influence. An important theoretical base for the recursive relations in recommender systems is provided by Reinforcing Spirals Theory (RST), which explains how there can be reciprocal relations between media selection, media use, and user dispositions (Slater, 2007, 2015). Thus, PMPF adds the concept of computational adaptability to RST and provides more detail on how user behaviors after previous exposures can become machine-readable inputs and influence recommender systems’ algorithms. Selective-exposure research provides an explanation of behavioral selection, echo-chamber theory highlights the relational aspects such as homophily and social reinforcement, while platform research draws attention to the computational aspect of visibility. PMPF adds the detail of how these concepts can become computationally coupled within recommender environments.

The computational coupling is central to the process of exposure. Users face a ranked informational landscape and react to it with such actions as clicking, viewing, dwelling, liking, sharing, or skipping. In case of PMPF, when such behaviors are captured and processed for future rankings, they can affect exposure conditions. The reviewed literature provides direct evidence of several types of return relationships between recommender, exposure, behavior, and relational states, although the exact form of recursion can vary among the reviewed works (Donkers, 2021; Wang, 2022; Gao, 2024; Duskin, 2024; Zheng, 2025). Thus, behavior becomes an action performed in response to an existing exposure environment and, under certain recommender system architecture, the input into the following personalization. Some other papers demonstrate the constituent mechanisms and theorize about feedback loops but do not provide evidence of return paths. Thus, PMPF combines evidence of different directness rather than claims that its complete recursive architecture is evidenced in all the reviewed literature.

Recursive relationship does not always lead to amplification of exposure. The following interactions may reproduce or amplify exposure patterns, but they may also attenuate, redirect or diversify them depending on the recommender system design, user actions and the context. The intervention-oriented studies explore how changes in recommendation design may affect diversity, homogeneity and serendipity and other exposure conditions (Sun, 2020; Wang, 2022; Gao, 2024; Treuillier, 2024; Zheng, 2025). Therefore, recursivity should be considered as return relationships between interaction states, rather than an inherently reinforcing process.

Relation processes also impact the interaction dynamics. Echo chamber effect differs from filter bubble effect, because homophily, clustering and social reinforcement deal with relational aspects of the informational circulation, while the filter bubble refers only to the diversity of recommended content. Relational structure may precede recommendation, interact with it, or change along with it. The studies of relational recommendations provide evidence of relations between recommender processes, homophily, clustering, and exposure conditions (Grossetti, 2021; Tommasel, 2021; De Biasio, 2023; Duskin, 2024; Yu, 2025). However, homophily or clustering alone cannot prove the existence of recursive reinforcement. The relevance of PMPF lies in the demonstration that recommendation is conducted in the social environments affecting informational circulation and engagement.

Such differences help to understand why the results on polarization were heterogeneous. Although exposure asymmetry, filter bubbles, echo chambers and polarization are related phenomena, they are not interchangeable and not necessarily a part of a fixed causal sequence. The changes in visibility and exposure diversity are about the informational conditions created by recommendation, while attitudinal or ideological polarization is another downstream outcome requiring different behaviors, relational conditions, and context. The studies on polarization examine different kinds of outcomes across different designs and platform contexts, some results show the context-dependent effects (Ludwig, 2023; Nechushtai, 2024; Shin, 2024; Ludwig, 2025; Schellingerhout, 2025). The methodological design also impacts the kind of evidential leverage researchers have: simulation studies analyze possible trajectories under certain assumptions, experimental studies provide evidences about causal relations in bounded circumstances, and audits identify recommendation/exposure patterns without proving attitudinal changes. Thus, the variations in the results across these designs should be interpreted in regard to evidentiary leverage of each method, not the contradiction.

Time is important, since recursivity involves successive interaction states, in agreement with the theories of reciprocal relationships between media use and user dispositions over time (Slater, 2007, 2015). However, the evidence provided in the current review does not suggest that there is a universal progression from repetitive exposures to attitudinal lock-ins. Limited longitudinal studies in real-world conditions prevent the conclusions about the persistence of the recursive exposure patterns and their consequences. The temporal development, therefore, should be considered as an empirical question, not a characteristic of the framework. The studies on misinformation and radicalization demonstrate the same dependence on the context. The studies indicate that recommendation is associated with the amplification, steering or mitigation of misinformation, conspiratorial materials, and other kinds of harmful content (Valdez, 2020; Pathak, 2023; Shin, 2024; Schellingerhout, 2025; Zhang, 2025). However, the reviewed literature does not prove that acceptance of misinformation or radicalization is inherent consequences of the recommendation. Exposure to harmful information, its acceptance, and ideological/behavioral change after are analytically different phenomena. PMPF treats informational context as a condition that affects the consequences of recommender-mediated exposure.

Such conditional approach makes clearer the central question of consensus and conflict in the paper. Consensus receives relatively little direct investigation in the reviewed literature, and its lack of presence should not be interpreted as the evidence that recommender systems cannot generate common informational environments. What the evidence clearly shows is that recommendation is able to affect the visibility, diversity, and distribution of informational opportunities within which the following interactions occur (Lunardi, 2020; Vrijenhoek, 2021; Michiels, 2023; Wang, 2025). Whether these conditions result in convergence, differentiation, fragmentation or polarization depends on the recommender system design, user behavior, relational structure, informational context, and their interaction over time. The theoretical question, thus, is not about whether recommender systems inherently create consensus or conflict, but about the conditions under which recommender-mediated exposure leads to different trajectories.

However, implications for platform governance go even beyond those effects that have been directly established in the course of the review. PMPF offers a basis for intervention beyond not only consideration of what type of content is shown but also of the processes of generation and modification of recommendation conditions as an object of intervention. Recommender designs aiming at diversity and serendipity represent the types of intervention objects considered in the literature (Sun, 2020; Wang, 2022; Gao, 2024; Treuillier, 2024; Zheng, 2025). However, their downstream impact on polarization has yet to be confirmed and requires empirical evaluation. In general terms, the normative concern does not lie in informational difference itself but in whether processes of recommendation mediate engagement with it. As a result, recursive exposure conditions may become an object of regulatory and democratic scrutiny without being assumed to lead to fragmentation (van Dijck et al., 2018).

There are some important limitations to these conclusions. Firstly, the chosen 2020–2025 publication period allows viewing this synthesis as a survey of contemporary evidence but not as a historical one. Secondly, methodological diversity leads to the fact that various forms of evidence (experiments, simulations, audits, systems design, qualitative data, and empirical models) are used. Demonstrations of complete recursive exposure pathways are mostly confined to certain types of studies, whereas longitudinal real-life evidence is not sufficient to make any conclusions regarding persistence of recursive exposure and its long-term consequences on attitudes. Additionally, the chosen search strategy and database limitations may have influenced the formation of this corpus as the primary search strategy relied on filter bubbles, echo chambers, and selective exposure, and additional analysis was done with regard to polarization in the process of sensitivity search. Therefore, studies using other terminology were possibly missed out. Finally, insufficient investigation of consensus prevents any comparison between convergence and conflict-related outcomes.

Thus, the outlined limitations allow forming a clear research agenda. First of all, future studies should go beyond isolated recommendation outputs and immediate behavioral responses and adopt designs able to observe successive states of interaction. This may include longitudinal platform studies, repeated-interaction experiments, computational audits taking into account behavioral feedback loops, and mixed-method designs. These kinds of studies will allow investigating whether and how behavioral responses affect subsequent recommendation and consequently evolution of exposure conditions. Distinguishing between recommendation outputs, exposure, user engagement, reinforcement of relational connections, and attitudinal outcomes is also required in order to test the proposed return pathways and explore conditions of their influence. In addition, greater focus on cross-cutting exposure, pluralistic information, and convergence in the literature is necessary due to current overemphasis on conflict-related outcomes.

In conclusion, PMPF may be viewed as a theoretical framework for the problem of computational adaptation. By describing how behavioral responses to the previous exposure may become inputs of subsequent ranking and personalization, PMPF offers the theoretical concept of recommender-mediated exposure as a potentially recursive phenomenon without assumptions about reinforcement and fragmentation of it. Consequently, PMPF should be perceived not as an already validated causal model but as a system-level theoretical framework generating testable hypotheses about recursive exposure pathways.

## 5 Conclusion

In summary, this review aimed to examine whether algorithmically organized recommendation contributes to common informational conditions or fragmentation and polarization. Synthesis of 60 studies shows that there is no simple answer either in terms of consensus or conflict. The evidence is more extensive and more direct in relation to informational exposure, while evidence regarding recursive relationships and polarization is more varied. Therefore, recommender systems should be considered as socio-technologies that configure the conditions of communication, but not mechanisms producing polarization per se. One of the implications of the review is that exposure conditions and polarization outcomes should be treated separately. Recommendation can influence the visibility, diversity, and distribution of recommended content, and, in some cases, the exposure of users to information. However, differences in exposure cannot be considered as polarization itself. Filter bubbles indicate certain patterns of narrowed or concentrated exposure; echo chambers concern the relations based on homophily, clustering, and social reinforcement; and polarization indicates the structured divergence in attitudes, affective orientations, perception, or ideology. In this sense, the evidence collected does not suggest the inevitability of the chain in which recommendation produces filter bubbles and then echo chambers and, finally, polarization.

On the contrary, the key theoretical contribution of the study to the field is the Platform-Mediated Polarization Framework (PMPF). PMPF is based on the recursive assumptions of Reinforcing Spirals Theory and integrates the evidence from selective exposure, echo chambers, recommender systems, and platform research. Its unique focus is the computational adaptation of informational environments. PMPF does not intend to replace other theories nor assume that recommender systems are self-reinforcing. On the contrary, PMPF shows that through recommendation, algorithmic processes, and social relations can become computationally integrated and form the loops in which previous exposures affect subsequent informational conditions. When responses to previous exposures are involved into the subsequent ranking and personalization of recommendations, these responses may contribute to constructing conditions of new exposure. The reviewed evidence includes such return pathways directly or indirectly; some papers show the components of these pathways or discuss the feedback without specifying the return pathway itself. Consequently, PMPF integrates varied evidence that is provided in the reviewed papers without making a claim that this framework as a whole has already been empirically verified.

This specification is necessary since recursion is not equivalent to amplification. Depending on the recommender architecture, user behavior, network structure, informational context, and temporal conditions, the recursive recommender-mediated processes can either reinforce existing trajectories, or diversify, redirect them. In this sense, the heterogeneous evidence regarding polarization can be interpreted. Experiments, simulations, audits, design studies, and observational research investigate different aspects of these processes and provide varied evidentiary power. Consequently, the variations in their findings should be explained not by contradictions but by different mechanisms, outcomes, platforms, and methods used. A similar conditional interpretation is also applicable to the relationship between misinformation and radicalization, where encountering, adopting, and subsequent changes in ideology or behavior are different analytically distinguishable outcomes.

Consequently, the findings also shift the focus of the paper’s problem from the question of consensus versus conflict to identification of conditions under which different communication trajectories emerge. The research in the corpus provides much more direct evidence on differentiated exposure and conflict-related outcomes than on consensus formation. This imbalance should not be interpreted as the evidence of necessity of the weakening of consensus or preference to polarization by recommender systems. Instead, it reveals the important limitation of the literature: the recommendation organizes the informational conditions in which convergence, difference, fragmentation, or polarization can emerge. The trajectory of the interaction depends on the combination of recommendation design, user behavior, network relations, informational context, and temporal development. Thus, the synthesis of the review shifts the problem from choosing between consensus and conflict to defining the conditions under which different trajectories emerge.

There are also implications for platform governance although they are normative rather than effect observed in the reviewed studies. Studies of diversity, serendipity, cross-cutting recommendation, and other related interventions show that recommender design can be used as an intervention point to modify the conditions of exposure. From the perspective of PMPF, therefore, the governance of the platform should also pay attention to processes of production and alteration of informational conditions. Whether these interventions can reduce polarization remains the empirical question. However, the conclusions of the study should be understood within its boundaries. The focus on publications from 2020 to 2025 allows synthesizing the current state of the literature rather than giving a complete history of recommender systems research. The use of Scopus and Web of Science databases might also mean that the relevant literature indexed elsewhere has been missed. Additionally, heterogeneity in methods restricts the comparability of study designs. Finally, the recursive return pathways have been identified directly in the subset of reviewed studies and the evidence from the real environment has been lacking. Therefore, the current literature does not allow making confident claims about persistence of the recursion and its long-term attitudinal effects.

These limitations define a more focused empirical agenda. Future research should move beyond consideration of single recommendation outputs and short-term responses to the designs allowing observing the interaction in several states. Longitudinal studies of platforms, repeated interaction experiments, and audits including the behavioral feedback could directly test how the user responses modify the conditions of the recommendation. This research should preserve the distinction between recommendation outputs, realized exposure, selective engagement, network reinforcement, and attitudinal outcomes. Comparative cross-platform research will help to determine how different architectures and informational conditions influence these processes. Finally, the convergence, cross-cutting exposure, and informational pluralism should receive more attention so that consensus and conflict could be considered as alternatives rather than assumed polarization as the expected end of algorithmically mediated communication. Thus, the significance of recommender systems is not in the necessity of determination of consensus or conflict, but rather in configuring the conditions in which such outcomes can emerge. PMPF considers this process as potentially recursive without specifying the direction and consequences of the recursion as an empirical issue. Therefore, the central problem of the future research is the identification of conditions of emergence of convergence, difference, fragmentation, or polarization in recursive exposure production.
