# Supplementary material for: Consensus or conflict? Recommender systems as recursive exposure systems in the production of polarization
Source: Front Sociol. 2026 Sep 30;11:1940431. doi: 10.3389/fsoc.2026.1940431 (PMC13662418; doi:10.3389/fsoc.2026.1940431)
Supplement: Supplementary file 1 [file Table_1.DOCX]

**Supplementary Table 1 . Study-Level Methodological Quality Appraisal**

Each study was assessed across six domains using a review-specific, design-sensitive rubric. Domains were scored as 0 (weak), 1 (adequate), or 2 (strong), yielding a total score from 0 to 12. For descriptive comparison, scores of 10–12 were categorized as higher methodological quality, 7–9 as moderate methodological quality, and 0–6 as lower methodological quality. These categories were used as review-specific descriptive indicators rather than externally validated quality thresholds or exclusion criteria.

*Domains: D1 = appropriateness of research design; D2 = adequacy of sample, dataset, or empirical basis; D3 = transparency and rigor of data collection, experimental procedures, or model construction; D4 = appropriateness and transparency of analysis; D5 = conceptual and operational clarity; D6 = alignment between evidence and claims.*

| **No.** | **Study** | **Year** | **Method** | **D1** | **D2** | **D3** | **D4** | **D5** | **D6** | **Total** | **Quality** |
| --- | --- | --- | --- | --- | --- | --- | --- | --- | --- | --- | --- |
| 1 | Aridor, G — Deconstructing the Filter Bubble: User Decision-Making and Recommender Systems | 2020 | Modeling / simulation | 2 | 1 | 2 | 2 | 2 | 2 | 11 | High |
| 2 | Joris, G. — News diversity and recommendation systems: Setting the interdisciplinary scene | 2020 | Modeling / simulation | 1 | 1 | 1 | 1 | 2 | 2 | 8 | Moderate |
| 3 | Lunardi, GM — A metric for Filter Bubble measurement in recommender algorithms considering the news domain | 2020 | Measurement / empirical modeling | 2 | 1 | 2 | 2 | 2 | 2 | 11 | High |
| 4 | Sun, J. — Leveraging cross domain recommendation models to alleviate filter bubble problems | 2020 | Experiment | 2 | 1 | 2 | 2 | 2 | 1 | 10 | High |
| 5 | Valdez, AC — Human and Algorithmic Contributions to Misinformation Online - Identifying the Culprit | 2020 | Measurement / empirical modeling | 2 | 1 | 1 | 2 | 2 | 1 | 9 | Moderate |
| 6 | Vrijenhoek, S — Pitch Proposal: Recommenders with a Mission - Assessing Diversity in News Recommendations | 2020 | Measurement / empirical modeling | 1 | 0 | 1 | 1 | 1 | 1 | 5 | Low |
| 7 | Donkers, T — The Dual Echo Chamber: Modeling Social Media Polarization for Interventional Recommending | 2021 | Modeling / simulation | 2 | 0 | 2 | 2 | 2 | 2 | 10 | High |
| 8 | Grossetti, Q — Reducing the filter bubble effect on Twitter by considering communities for recommendations | 2021 | System / algorithm design | 2 | 1 | 2 | 2 | 1 | 2 | 10 | High |
| 9 | Morales, PR — Auditing the Effect of Social Network Recommendations on Polarization in Geometrical Ideological Spaces | 2021 | Audit | 2 | 1 | 2 | 2 | 2 | 2 | 11 | High |
| 10 | Tommasel, A — I Want to Break Free! Recommending Friends from Outside the Echo Chamber | 2021 | System / algorithm design | 2 | 1 | 2 | 2 | 1 | 2 | 10 | High |
| 11 | Vrijenhoek, S — Recommenders with a Mission: Assessing Diversity in News Recommendations | 2021 | Measurement / empirical modeling | 2 | 1 | 2 | 2 | 2 | 2 | 11 | High |
| 12 | Zhao, X — Rabbit Holes and Taste Distortion: Distribution-Aware Recommendation with Evolving Interests | 2021 | System / algorithm design | 2 | 1 | 2 | 2 | 1 | 2 | 10 | High |
| 13 | El-Moutaouakkil, Z — Polarization in Personalized Recommendations: Balancing Safety and Accuracy | 2022 | Modeling / simulation | 2 | 1 | 2 | 2 | 1 | 1 | 9 | Moderate |
| 14 | Gao, ZL — Mitigating the Filter Bubble while Maintaining Relevance: Targeted Diversification with VAE-based Recommender Systems | 2022 | Experiment | 2 | 1 | 2 | 2 | 2 | 1 | 10 | High |
| 15 | Han, H — SSLE: A framework for evaluating the Filter Bubble effect on the news aggregator and recommenders | 2022 | Modeling / simulation | 2 | 1 | 2 | 2 | 2 | 2 | 11 | High |
| 16 | Sonoda, A. — Improving Recommendation Diversity by Maximizing Semantic Volume | 2022 | Experiment | 2 | 1 | 2 | 2 | 1 | 1 | 9 | Moderate |
| 17 | Stray, J. — Designing recommender systems to depolarize | 2022 | Measurement / empirical modeling | 1 | 1 | 2 | 1 | 2 | 1 | 8 | Moderate |
| 18 | Wang, WJ — User-controllable Recommendation Against Filter Bubbles | 2022 | Experiment | 2 | 1 | 2 | 2 | 2 | 1 | 10 | High |
| 19 | Areeb, QM — Filter bubbles in recommender systems: Fact or fallacy-A systematic review | 2023 | Review / bibliometric | 2 | 1 | 2 | 2 | 2 | 1 | 10 | High |
| 20 | De Biasio, A — On the problem of recommendation for sensitive users and influential items: Simultaneously maintaining interest and diversity | 2023 | System / algorithm design | 2 | 1 | 2 | 2 | 1 | 2 | 10 | High |
| 21 | Du, Y.R. — Personalization, Echo Chambers, News Literacy, and Algorithmic Literacy: A Qualitative Study of AI-Powered News App Users | 2023 | Qualitative study | 2 | 0 | 2 | 2 | 2 | 2 | 10 | High |
| 22 | Knudsen, E. — Modeling news recommender systems’ conditional effects on selective exposure: evidence from two online experiments | 2023 | Experiment | 2 | 2 | 2 | 2 | 2 | 2 | 12 | High |
| 23 | Lieto, A. — DEGARI 2.0: A diversity-seeking, explainable, and affective art recommender for social inclusion | 2023 | Modeling / simulation | 2 | 1 | 2 | 1 | 1 | 1 | 8 | Moderate |
| 24 | Ludwig, K. — Divided by the Algorithm? The (Limited) Effects of Content- and Sentiment-Based News Recommendation on Affective, Ideological, and Perceived Polarization | 2023 | Experiment | 2 | 2 | 2 | 2 | 2 | 2 | 12 | High |
| 25 | Makhortykh, M. — Can Filter Bubbles Protect Information Freedom? Discussions of Algorithmic News Recommenders in Eastern Europe | 2023 | System / algorithm design | 2 | 1 | 2 | 1 | 2 | 1 | 9 | Moderate |
| 26 | Michiels, L — How ShouldWe Measure Filter Bubbles? A Regression Model and Evidence for Online News | 2023 | Modeling / simulation | 2 | 1 | 2 | 2 | 2 | 2 | 11 | High |
| 27 | Pathak, R — Understanding the Contribution of Recommendation Algorithms on Misinformation Recommendation and Misinformation Dissemination on Social Networks | 2023 | Modeling / simulation | 2 | 1 | 2 | 2 | 2 | 2 | 11 | High |
| 28 | Zhang, H — Evolution of Filter Bubbles and Polarization in News Recommendation | 2023 | Experiment | 2 | 1 | 2 | 2 | 2 | 1 | 10 | High |
| 29 | Duskin, K — Echo Chambers in the Age of Algorithms: An Audit of Twitter's Friend Recommender System | 2024 | Experiment | 2 | 1 | 2 | 2 | 2 | 2 | 11 | High |
| 30 | Gao, CM — CIRS: Bursting Filter Bubbles by Counterfactual Interactive Recommender System | 2024 | Experiment | 2 | 1 | 2 | 2 | 2 | 1 | 10 | High |
| 31 | Jeng, JH — Bridging Viewpoints in News with Recommender Systems | 2024 | Measurement / empirical modeling | 2 | 1 | 2 | 2 | 1 | 1 | 9 | Moderate |
| 32 | Jiang, T. — Breaking the Filter Bubbles: The Effect of Algorithm Curation on Selectivity of Information Consumption and Attitude Extremity; 应对过滤气泡: 算法策展对用户信息消费行为选择性和态度极端化的影响 | 2024 | Measurement / empirical modeling | 2 | 1 | 2 | 2 | 2 | 1 | 10 | High |
| 33 | Lechiakh, M. — Towards long-term depolarized interactive recommendations | 2024 | Experiment | 2 | 1 | 2 | 2 | 2 | 2 | 11 | High |
| 34 | Nechushtai, E. — More of the Same? Homogenization in News Recommendations When Users Search on Google, YouTube, Facebook, and Twitter | 2024 | Audit | 2 | 2 | 2 | 2 | 2 | 2 | 12 | High |
| 35 | Pathak, R — Analyzing the Interplay between Diversity of News Recommendations and Misinformation Spread in Social Media | 2024 | Modeling / simulation | 2 | 0 | 2 | 2 | 2 | 2 | 10 | High |
| 36 | Pathak, R. — Advancing Misinformation Awareness in Recommender Systems for Social Media Information Integrity | 2024 | System / algorithm design | 2 | 1 | 2 | 2 | 1 | 1 | 9 | Moderate |
| 37 | Reid, J. — Digitising “The Big Lie”: Algorithmic Curation as an Inhibitor of Media Exposure Diversity Online | 2024 | Modeling / simulation | 1 | 1 | 1 | 1 | 2 | 1 | 7 | Moderate |
| 38 | Sergas, U — Perspective Diversification News Recommender System | 2024 | Modeling / simulation | 2 | 1 | 2 | 2 | 1 | 1 | 9 | Moderate |
| 39 | Shin, D. — How Algorithms Promote Self-Radicalization: Audit of TikTok’s Algorithm Using a Reverse Engineering Method | 2024 | Audit | 2 | 1 | 2 | 2 | 2 | 2 | 11 | High |
| 40 | Sonoda, A — User Experiments on the Effect of the Diversity of Consumption on News Services | 2024 | Experiment | 2 | 1 | 2 | 2 | 2 | 1 | 10 | High |
| 41 | Treuillier, C — Beyond Trade-offs: Unveiling Fairness-Constrained Diversity in News Recommender Systems | 2024 | Experiment | 2 | 1 | 2 | 2 | 2 | 1 | 10 | High |
| 42 | Xu, YB — GS2-RS: A Generative Approach for Alleviating ColdStart and Filter Bubbles in Recommender Systems | 2024 | Experiment | 2 | 1 | 2 | 2 | 1 | 1 | 9 | Moderate |
| 43 | Zhou, X. — "Bee and I need diversity!" Break Filter Bubbles in Recommendation Systems through Embodied AI Learning | 2024 | System / algorithm design | 2 | 1 | 2 | 2 | 1 | 1 | 9 | Moderate |
| 44 | Ahmmad, M. — Trap of Social Media Algorithms: A Systematic Review of Research on Filter Bubbles, Echo Chambers, and Their Impact on Youth | 2025 | Review / bibliometric | 2 | 1 | 2 | 1 | 2 | 1 | 9 | Moderate |
| 45 | Alam, MJ — Combating Echo Chambers in Online Social Network by Increasing Content Diversity in Recommendation | 2025 | Experiment | 2 | 1 | 1 | 1 | 1 | 1 | 7 | Moderate |
| 46 | Bojic, L — An Agent-Based Simulation of Politicized Topics Using Large Language Models: Algorithmic Personalization and Polarization on Social Media | 2025 | Qualitative study | 2 | 0 | 2 | 2 | 2 | 2 | 10 | High |
| 47 | Han, G — Controlling Diversity at Inference: Guiding Diffusion Recommender Models with Targeted Category Preferences | 2025 | Experiment | 2 | 1 | 2 | 2 | 1 | 1 | 9 | Moderate |
| 48 | Jiang, T. — Restraining the formation of filter bubbles with algorithmic affordances: Toward more balanced information consumption and decreased attitude extremity | 2025 | Experiment | 2 | 2 | 2 | 2 | 2 | 2 | 12 | High |
| 49 | Kim, S — Balancing Diversity in Session-Based Recommendation Between Relevance and Unexpectedness | 2025 | Experiment | 2 | 1 | 2 | 2 | 1 | 1 | 9 | Moderate |
| 50 | Ludwig, K. — Putting ‘filter bubble’ effects to the test: evidence on the polarizing impact of ideology-based news recommendation from two experiments in Germany and the U.S. | 2025 | Experiment | 2 | 2 | 2 | 2 | 2 | 2 | 12 | High |
| 51 | Pathak, R — Circumventing Misinformation Controls: Assessing the Robustness of Intervention Strategies in Recommender Systems | 2025 | Modeling / simulation | 2 | 0 | 2 | 2 | 2 | 2 | 10 | High |
| 52 | Poechhacker, N. — Recommender Systems beyond the Filter Bubble: Algorithmic Media and the Fabrication of Publics | 2025 | System / algorithm design | 1 | 0 | 2 | 1 | 1 | 1 | 6 | Low |
| 53 | Ren, BF — Leveraging personalized diversity level for recommendations with knowledge graph | 2025 | Experiment | 2 | 1 | 2 | 2 | 1 | 1 | 9 | Moderate |
| 54 | Schellingerhout, R. — Accounting for Personalization in Personalization Algorithms: YouTube’s Treatment of Conspiracy Content | 2025 | Experiment | 2 | 1 | 2 | 2 | 2 | 2 | 11 | High |
| 55 | Wang, R. — How News Recommendation System Effects User’s Individual and Aggregate Topic Diversity—A Study of Simulation Analysis | 2025 | Modeling / simulation | 2 | 1 | 2 | 2 | 2 | 2 | 11 | High |
| 56 | Xi, YJ — Bursting Filter Bubble: Enhancing Serendipity Recommendations with Aligned Large Language Models | 2025 | Experiment | 2 | 1 | 2 | 2 | 1 | 1 | 9 | Moderate |
| 57 | Yan, C. — Knowledge mapping of information cocoons: A bibliometric study using visual analysis | 2025 | Review / bibliometric | 2 | 1 | 2 | 2 | 2 | 1 | 10 | High |
| 58 | Yu, DN — Breaking the Loop: Causal Learning to Mitigate Echo Chambers in Social Networks | 2025 | Experiment | 2 | 1 | 2 | 2 | 2 | 2 | 11 | High |
| 59 | Zhang, MY — How Misinformation Diffuses on Online Social Networks: Radical Opinions, Adaptive Relationship, and Algorithmic Intervention | 2025 | Experiment | 2 | 1 | 2 | 2 | 2 | 2 | 11 | High |
| 60 | Zheng, Y. — HyperCRS: Hypergraph-Aware Multi-Grained Preference Learning to Burst Filter Bubbles in Conversational Recommendation System | 2025 | Modeling / simulation | 2 | 1 | 2 | 2 | 1 | 1 | 9 | Moderate |

**Summary of appraisal results.** Among the 60 included studies, 38 were classified as higher methodological quality, 20 as moderate methodological quality, and two as lower methodological quality. The mean methodological quality score was 9.8 out of 12.

*Note. Methodological quality was assessed separately from the directness of evidence for recursive dynamics and polarization-related outcomes. A higher methodological-quality score therefore does not necessarily indicate direct evidence of recursivity or polarization.*
