# Supplementary material for: Consensus or conflict? Recommender systems as recursive exposure systems in the production of polarization
Source: Front Sociol. 2026 Sep 30;11:1940431. doi: 10.3389/fsoc.2026.1940431 (PMC13662418; doi:10.3389/fsoc.2026.1940431)
Supplement: Supplementary file 2 [file Table_2.DOCX]

**Supplementary Table S2. Study-level evidence map of the 60 studies included in the synthesis**

*The table maps the evidentiary role of each included study. “Recursive evidence” follows the relationship-based classification in Section 2.8; “Quality” follows the review-specific appraisal in Section 2.7.*

| **Study** | **Platform / context** | **Design** | **Primary construct** | **Mechanism examined** | **Level of analysis** | **Principal outcome** | **Recursive evidence** | **Quality** |
| --- | --- | --- | --- | --- | --- | --- | --- | --- |
| Aridor G (2020) | General recommender systems | Modeling / simulation | Filter bubbles / cocoons | Exposure diversity / diversification | Algorithmic / system | Links recommendation design to exposure diversity/bubble-related outcomes. | Partial | Higher |
| Joris G. (2020) | News / news recommender | Conceptual / interdisciplinary synthesis | Filter bubbles / cocoons | Exposure diversity / diversification | Algorithmic / system | Establishes an interdisciplinary framework for assessing news diversity and recommendation systems; does not directly test recommender effects. | Conceptual | Moderate |
| Lunardi GM (2020) | News app | Measurement / empirical modeling | Filter bubbles / cocoons | Exposure diversity / diversification | System / exposure | Proposes/uses measurement of filter-bubble effects. | Partial | Higher |
| Sun J. (2020) | Social media | Experiment | Filter bubbles / cocoons | Exposure diversity / diversification | Behavioural / exposure | Evaluates cross-domain recommendation as a strategy to alleviate filter bubbles while preserving recommendation relevance. | Conceptual | Higher |
| Valdez AC (2020) | News / news recommender | Measurement / empirical modeling | Filter bubbles / cocoons | Misinformation / radicalization pathway | System / exposure | Examines how human behaviour and algorithmic processes jointly contribute to online misinformation and its amplification. | Conceptual | Moderate |
| Vrijenhoek S (2020) | News / news recommender | Conceptual / project proposal | Filter bubbles / cocoons | Exposure diversity / diversification | System / exposure | Proposes a framework and diversity metrics for assessing normative diversity in news recommendations; reports an ongoing project rather than completed effects. | Conceptual | Lower |
| Donkers T (2021) | Social media | Modeling / simulation | Polarization | Network clustering / reinforcement | Relational / system | Models echo-chamber dynamics and polarization trajectories. | Direct | Higher |
| Grossetti Q (2021) | Twitter/X | System / algorithm design | Filter bubbles / cocoons | Network clustering / reinforcement | Relational / system | Finds network ranking can reinforce homophily; diversity can counter it. | Partial | Higher |
| Morales PR (2021) | Twitter/X | Audit | Polarization | Network recommendation / polarization | System / exposure | Audits how social-network recommendations can alter ideological structure and polarization in modeled ideological spaces. | Direct | Higher |
| Tommasel A (2021) | Twitter/X | System / algorithm design | Filter bubbles / cocoons | Network clustering / reinforcement | Relational / system | Develops friend recommendations that connect users outside their echo chambers while accounting for user interests. | Partial | Higher |
| Vrijenhoek S (2021) | News / news recommender | Measurement / empirical modeling | Filter bubbles / cocoons | Exposure diversity / diversification | System / exposure | Finds changes in viewpoint/topic diversity or polarization-related exposure. | Conceptual | Higher |
| Zhao X (2021) | Movie / media recommender | System / algorithm design | Filter bubbles / cocoons | Exposure diversity / diversification | Algorithmic / system | Links recommendation design to exposure diversity/bubble-related outcomes. | Partial | Higher |
| El-Moutaouakkil Z (2022) | General recommender systems | Modeling / simulation | Polarization | Exposure diversity / diversification | Algorithmic / system | Models the trade-off between recommendation accuracy and limiting polarization in personalized recommendation. | None | Moderate |
| Gao ZL (2022) | General recommender systems | Experiment | Filter bubbles / cocoons | Exposure diversity / diversification | Behavioural / exposure | Tests targeted diversification to mitigate filter bubbles while maintaining recommendation relevance. | None | Higher |
| Han H (2022) | News aggregators / recommenders | Empirical evaluation / longitudinal audit | Filter bubbles / cocoons | Exposure feedback / filter-bubble evolution | System / exposure | Evaluates filter-bubble evolution in personalized news recommendation, showing convergence between user interests and recommended content over repeated interactions. | Direct | Higher |
| Sonoda A. (2022) | News / news recommender | Experiment | Filter bubbles / cocoons | Network clustering / reinforcement | Relational / behavioural | Supports diversity/serendipity design against homogenization. | None | Moderate |
| Stray J. (2022) | Social media | Conceptual / design synthesis | Polarization | Exposure diversity / diversification | System / exposure | Synthesizes design approaches for recommender systems intended to reduce polarization and identifies relevant intervention objectives. | Conceptual | Moderate |
| Wang WJ (2022) | General recommender systems | Experiment | Filter bubbles / cocoons | Exposure diversity / diversification | Behavioural / exposure | Links recommendation design to exposure diversity/bubble-related outcomes. | Direct | Higher |
| Areeb QM (2023) | Social media | Review / bibliometric | Filter bubbles / cocoons | Network clustering / reinforcement | Multi-level | Maps themes, measures, and gaps in bubbles/chambers/polarization research. | Conceptual | Higher |
| De Biasio A (2023) | Social network | System / algorithm design | Echo chambers | Network clustering / reinforcement | Relational / system | Supports diversity/serendipity design against homogenization. | Partial | Higher |
| Du Y.R. (2023) | News app | Qualitative study | Echo chambers | Network clustering / reinforcement | User / interpretive | Shows how users interpret personalization and echo chambers in AI-powered news apps, highlighting the roles of news and algorithmic literacy. | Partial | Higher |
| Knudsen E. (2023) | News / news recommender | Experiment | Filter bubbles / cocoons | Selective engagement / exposure | Behavioural / exposure | Selective-exposure effects vary by prior ideology and use conditions. | None | Higher |
| Lieto A. (2023) | Art recommender | Modeling / simulation | Filter bubbles / cocoons | Exposure diversity / diversification | Algorithmic / system | Supports diversity/serendipity design against homogenization. | None | Moderate |
| Ludwig K. (2023) | News / news recommender | Experiment | Polarization | Polarization pathway | Behavioural / exposure | Finds limited and conditional effects of news recommendation on affective, ideological, and perceived polarization. | None | Higher |
| Makhortykh M. (2023) | News / news recommender | Document analysis | Filter bubbles / cocoons | Exposure diversity / diversification | Institutional / discursive | Examines how algorithmic news recommenders and filter bubbles are discussed in Eastern European information-freedom debates. | Conceptual | Moderate |
| Michiels L (2023) | News / news recommender | Modeling / simulation | Filter bubbles / cocoons | Exposure diversity / diversification | Algorithmic / system | Proposes/uses measurement of filter-bubble effects. | Partial | Higher |
| Pathak R (2023) | Twitter/X | Modeling / simulation | Filter bubbles / cocoons | Misinformation / radicalization pathway | Relational / system | Examines misinformation amplification and possible mitigation. | Partial | Higher |
| Zhang H (2023) | News / news recommender | Modeling / simulation | Polarization | Exposure diversity / diversification | Algorithmic / system | Simulates the long-run evolution of filter bubbles and polarization under news recommendation. | Direct | Higher |
| Duskin K (2024) | Twitter/X | Algorithmic audit | Echo chambers | Network clustering / reinforcement | Relational / system | Audits Twitter's friend recommender and finds recommendation patterns capable of increasing network homogeneity and echo-chamber formation. | Direct | Higher |
| Gao CM (2024) | General recommender systems | Experiment | Filter bubbles / cocoons | Exposure diversity / diversification | Behavioural / exposure | Tests diversification intended to reduce homogeneity while retaining relevance. | Direct | Higher |
| Jeng JH (2024) | News / news recommender | Doctoral research / design proposal | Polarization | Exposure diversity / diversification | System / exposure | Proposes and motivates viewpoint-bridging news recommender interventions; the contribution describes a research program rather than completed polarization effects. | None | Moderate |
| Jiang T. (2024) | News / news recommender | Measurement / empirical modeling | Filter bubbles / cocoons | Selective consumption / attitude extremity | Behavioural / exposure | Examines how algorithmic curation affects selective information consumption and attitude extremity. | None | Higher |
| Lechiakh M. (2024) | General recommender systems | Experiment | Polarization | Exposure diversity / diversification | Behavioural / exposure | Tests diversification intended to reduce homogeneity while retaining relevance. | Direct | Higher |
| Nechushtai E. (2024) | Google / Google News / Facebook / YouTube / Twitter | Audit | Filter bubbles / cocoons | Exposure diversity / diversification | System / exposure | Finds substantial homogenization in top-ranked news recommendations across major search and social platforms. | None | Higher |
| Pathak R (2024) | Twitter/X | Modeling / simulation | Filter bubbles / cocoons | Misinformation / radicalization pathway | Relational / system | Examines misinformation amplification and possible mitigation. | None | Higher |
| Pathak R. (2024) | Twitter/X | System / algorithm design | Filter bubbles / cocoons | Misinformation / radicalization pathway | Relational / system | Examines misinformation amplification and possible mitigation. | Conceptual | Moderate |
| Reid J. (2024) | General recommender systems | Conceptual / theoretical analysis | Filter bubbles / cocoons | Exposure diversity / diversification | Algorithmic / system | Argues that algorithmic curation can constrain media-exposure diversity and thereby contribute to filter-bubble conditions. | Conceptual | Moderate |
| Sergas U (2024) | News / news recommender | System / algorithm design | Filter bubbles / cocoons | Network clustering / reinforcement | Relational / system | Develops a perspective-diversification news recommender intended to broaden viewpoint exposure; downstream polarization is not directly tested. | None | Moderate |
| Shin D. (2024) | TikTok | Audit | Polarization | Misinformation / radicalization pathway | System / exposure | Audit detects clustering, misinformation, or echo-chamber steering. | Direct | Higher |
| Sonoda A (2024) | News / news recommender | Experiment | Echo chambers | Network clustering / reinforcement | Relational / behavioural | Supports diversity/serendipity design against homogenization. | None | Higher |
| Treuillier C (2024) | News / news recommender | Experiment | Filter bubbles / cocoons | Exposure diversity / diversification | Behavioural / exposure | Evaluates fairness-constrained diversification in news recommendation, examining trade-offs among relevance, diversity, and fairness. | None | Higher |
| Xu YB (2024) | General recommender systems | Experiment | Filter bubbles / cocoons | Exposure diversity / diversification | Behavioural / exposure | Tests diversification intended to reduce homogeneity while retaining relevance. | None | Moderate |
| Zhou X. (2024) | General recommender systems | HCI / embodied AI intervention | Filter bubbles / cocoons | Exposure diversity / diversification | User / educational | Evaluates an embodied AI-literacy intervention designed to help users understand how repeated choices can contribute to filter bubbles. | None | Moderate |
| Ahmmad M. (2025) | YouTube | Review / bibliometric | Polarization | Network clustering / reinforcement | Multi-level | Maps themes, measures, and gaps in bubbles/chambers/polarization research. | Conceptual | Moderate |
| Alam MJ (2025) | Social network | Experiment | Echo chambers | Network clustering / reinforcement | Relational / behavioural | Evaluates whether increasing recommendation diversity can mitigate echo-chamber formation in a simulated social-network setting. | None | Moderate |
| Bojic L (2025) | Social media | Agent-based modeling / simulation | Polarization | Exposure diversity / diversification | Computational / multi-agent | Uses an LLM-based agent simulation in which personalized exposure and user responses recursively shape subsequent content, producing polarization under specified conditions. | Direct | Higher |
| Han G (2025) | General recommender systems | Experiment | Filter bubbles / cocoons | Exposure diversity / diversification | Behavioural / exposure | Supports diversity/serendipity design against homogenization. | None | Moderate |
| Jiang T. (2025) | General recommender systems | Experiment | Filter bubbles / cocoons | Exposure diversity / diversification | Behavioural / exposure | Links recommendation design to exposure diversity/bubble-related outcomes. | None | Higher |
| Kim S (2025) | General recommender systems | Experiment | Filter bubbles / cocoons | Exposure diversity / diversification | Behavioural / exposure | Supports diversity/serendipity design against homogenization. | None | Moderate |
| Ludwig K. (2025) | News / news recommender | Experiment | Polarization | Polarization pathway | Behavioural / exposure | Ideology-based news recommendation shows bounded, context-dependent polarization. | None | Higher |
| Pathak R (2025) | News / news recommender | Modeling / simulation | Filter bubbles / cocoons | Misinformation / radicalization pathway | Algorithmic / system | Examines misinformation amplification and possible mitigation. | None | Higher |
| Poechhacker N. (2025) | General recommender systems | System / algorithm design | Filter bubbles / cocoons | Exposure diversity / diversification | Algorithmic / system | Links recommendation design to exposure diversity/bubble-related outcomes. | Conceptual | Lower |
| Ren BF (2025) | General recommender systems | Experiment | Filter bubbles / cocoons | Exposure diversity / diversification | Behavioural / exposure | Supports diversity/serendipity design against homogenization. | Partial | Moderate |
| Schellingerhout R. (2025) | YouTube | Experiment | Filter bubbles / cocoons | Misinformation / radicalization pathway | Behavioural / exposure | YouTube personalization shapes diversity and may channel homogeneous/conspiratorial exposure. | Direct | Higher |
| Wang R. (2025) | News / news recommender | Modeling / simulation | Filter bubbles / cocoons | Exposure diversity / diversification | Algorithmic / system | Finds changes in viewpoint/topic diversity or polarization-related exposure. | Direct | Higher |
| Xi YJ (2025) | General recommender systems | Experiment | Filter bubbles / cocoons | Exposure diversity / diversification | Behavioural / exposure | Tests diversification intended to reduce homogeneity while retaining relevance. | Conceptual | Moderate |
| Yan C. (2025) | Information-cocoon literature | Review / bibliometric | Filter bubbles / information cocoons | Field structure / thematic development | Literature / field | Maps the intellectual structure, themes, and development of information-cocoon research through bibliometric analysis. | Conceptual | Higher |
| Yu DN (2025) | Social network | Experiment | Echo chambers | Network clustering / reinforcement | Relational / behavioural | Links recommendation design to exposure diversity/bubble-related outcomes. | Partial | Higher |
| Zhang MY (2025) | News / news recommender | Experiment | Polarization | Misinformation / radicalization pathway | Behavioural / exposure | Examines misinformation amplification and possible mitigation. | Direct | Higher |
| Zheng Y. (2025) | Conversational recommender | System / algorithm design | Filter bubbles / cocoons | Exposure diversity / diversification | Algorithmic / system | Develops and benchmarks a hypergraph-aware conversational recommender designed to improve preference diversity and mitigate filter-bubble effects. | Direct | Moderate |

*Note. Direct = direct recursive evidence; Partial = partial/component evidence; Conceptual = conceptual/theoretical recursivity; None = no recursive evidence. Levels of analysis are review-level analytical classifications derived from study design, platform/context, constructs, and reported outcomes. Quality categories are descriptive appraisal categories, not exclusion thresholds.*
