# Supplementary material for: Consensus or conflict? Recommender systems as recursive exposure systems in the production of polarization
Source: Front Sociol. 2026 Sep 30;11:1940431. doi: 10.3389/fsoc.2026.1940431 (PMC13662418; doi:10.3389/fsoc.2026.1940431)
Supplement: Supplementary file 3 [file Table_3.DOCX]

**Supplementary Table S3. Characteristics of the 60 Included Studies**

| **Author/year** | **Platform** | **Context** | **Research design** | **Data/sample** | **Concepts examined** | **Main finding** | **Study title** |
| --- | --- | --- | --- | --- | --- | --- | --- |
| Aridor, G (2020) | General recommender systems | General recommender-system design/evaluation | Modeling / simulation | Model/simulation-based evidence; specific dataset/sample size not captured in the supplied coding sheet. | Filter bubbles / cocoons | Addresses how recommendation design interacts with exposure diversity, filter bubbles, or polarization-related outcomes. | Deconstructing the Filter Bubble: User Decision-Making and Recommender Systems |
| Joris, G. (2020) | News / news recommender | News recommendation and online news exposure | Modeling / simulation | Model/simulation-based evidence; specific dataset/sample size not captured in the supplied coding sheet. | Filter bubbles / cocoons; Selective exposure; Exposure diversity | Synthesizes prior work and maps major themes, measures, and gaps around recommender-driven bubbles, chambers, or polarization. | News diversity and recommendation systems: Setting the interdisciplinary scene |
| Lunardi, GM (2020) | News app | AI-powered/news-app recommendation and news exposure | Measurement / empirical modeling | Empirical/measurement data; specific dataset/sample size not captured in the supplied coding sheet. | Filter bubbles / cocoons; Exposure diversity | Proposes a measurement framework for quantifying filter-bubble effects rather than assuming them. | A metric for Filter Bubble measurement in recommender algorithms considering the news domain |
| Sun, J. (2020) | Social media | Social-media recommendation and information exposure | Experiment | Experimental evidence; participant/dataset size not captured in the supplied coding sheet. | Filter bubbles / cocoons; Exposure diversity | Designs or tests diversification strategies intended to reduce homogeneity while preserving recommendation relevance. | Leveraging cross domain recommendation models to alleviate filter bubble problems |
| Valdez, AC (2020) | News / news recommender | News recommendation and online news exposure | Measurement / empirical modeling | Empirical/measurement data; specific dataset/sample size not captured in the supplied coding sheet. | Filter bubbles / cocoons; Misinformation / radicalization | Examines how recommendation logic can amplify misinformation spread and how interventions may reduce that risk. | Human and Algorithmic Contributions to Misinformation Online - Identifying the Culprit |
| Vrijenhoek, S (2020) | News / news recommender | News recommendation and online news exposure | Measurement / empirical modeling | Empirical/measurement data; specific dataset/sample size not captured in the supplied coding sheet. | Filter bubbles / cocoons; Selective exposure; Exposure diversity | Shows news recommendation systems materially affect viewpoint diversity, topic diversity, or polarization-related exposure patterns. | Pitch Proposal: Recommenders with a Mission - Assessing Diversity in News Recommendations |
| Donkers, T (2021) | Social media | Social-media recommendation and information exposure | Modeling / simulation | Model/simulation-based evidence; specific dataset/sample size not captured in the supplied coding sheet. | Polarization; Echo chambers; Exposure diversity | Models echo-chamber dynamics and evaluates how recommendation or network interventions alter polarization trajectories. | The Dual Echo Chamber: Modeling Social Media Polarization for Interventional Recommending |
| Grossetti, Q (2021) | Twitter/X | Twitter/X recommendation, exposure, and network dynamics | System / algorithm design | System/algorithm evaluation data; specific benchmark/sample size not captured in the supplied coding sheet. | Filter bubbles / cocoons; Echo chambers; Exposure diversity | Analyzes recommendation on Twitter/X and finds network-based ranking can reinforce homophily unless diversity is added. | Reducing the filter bubble effect on Twitter by considering communities for recommendations |
| Morales, PR (2021) | Twitter/X | Twitter/X recommendation, exposure, and network dynamics | Audit | Platform/audit observations; specific audit sample size not captured in the supplied coding sheet. | Polarization | Audits recommendation outputs to detect whether the system steers users toward ideological clustering, misinformation, or echo chambers. | Auditing the Effect of Social Network Recommendations on Polarization in Geometrical Ideological Spaces |
| Tommasel, A (2021) | Twitter/X | Twitter/X recommendation, exposure, and network dynamics | System / algorithm design | System/algorithm evaluation data; specific benchmark/sample size not captured in the supplied coding sheet. | Filter bubbles / cocoons; Echo chambers; Exposure diversity | Designs or tests diversification strategies intended to reduce homogeneity while preserving recommendation relevance. | I Want to Break Free! Recommending Friends from Outside the Echo Chamber |
| Vrijenhoek, S (2021) | News / news recommender | News recommendation and online news exposure | Measurement / empirical modeling | Empirical/measurement data; specific dataset/sample size not captured in the supplied coding sheet. | Filter bubbles / cocoons; Selective exposure; Exposure diversity | Shows news recommendation systems materially affect viewpoint diversity, topic diversity, or polarization-related exposure patterns. | Recommenders with a Mission: Assessing Diversity in News Recommendations |
| Zhao, X (2021) | Movie / media recommender | Movie/media recommendation and evolving preferences | System / algorithm design | System/algorithm evaluation data; specific benchmark/sample size not captured in the supplied coding sheet. | Filter bubbles / cocoons | Addresses how recommendation design interacts with exposure diversity, filter bubbles, or polarization-related outcomes. | Rabbit Holes and Taste Distortion: Distribution-Aware Recommendation with Evolving Interests |
| El-Moutaouakkil, Z (2022) | General recommender systems | General recommender-system design/evaluation | Modeling / simulation | Model/simulation-based evidence; specific dataset/sample size not captured in the supplied coding sheet. | Polarization | Addresses how recommendation design interacts with exposure diversity, filter bubbles, or polarization-related outcomes. | Polarization in Personalized Recommendations: Balancing Safety and Accuracy |
| Gao, ZL (2022) | General recommender systems | General recommender-system design/evaluation | Experiment | Experimental evidence; participant/dataset size not captured in the supplied coding sheet. | Polarization; Filter bubbles / cocoons; Exposure diversity | Designs or tests diversification strategies intended to reduce homogeneity while preserving recommendation relevance. | Mitigating the Filter Bubble while Maintaining Relevance: Targeted Diversification with VAE-based Recommender Systems |
| Han, H (2022) | Twitter/X | Twitter/X recommendation, exposure, and network dynamics | Modeling / simulation | Model/simulation-based evidence; specific dataset/sample size not captured in the supplied coding sheet. | Polarization; Filter bubbles / cocoons | Proposes a measurement framework for quantifying filter-bubble effects rather than assuming them. | SSLE: A framework for evaluating the Filter Bubble effect on the news aggregator and recommenders |
| Sonoda, A. (2022) | News / news recommender | News recommendation and online news exposure | Experiment | Experimental evidence; participant/dataset size not captured in the supplied coding sheet. | Filter bubbles / cocoons; Echo chambers; Exposure diversity | Emphasizes diversity- or serendipity-oriented design as a way to counter homogenization in recommendations. | Improving Recommendation Diversity by Maximizing Semantic Volume |
| Stray, J. (2022) | Social media | Social-media recommendation and information exposure | Measurement / empirical modeling | Empirical/measurement data; specific dataset/sample size not captured in the supplied coding sheet. | Polarization; Filter bubbles / cocoons; Exposure diversity | Designs or tests diversification strategies intended to reduce homogeneity while preserving recommendation relevance. | Designing recommender systems to depolarize |
| Wang, WJ (2022) | General recommender systems | General recommender-system design/evaluation | Experiment | Experimental evidence; participant/dataset size not captured in the supplied coding sheet. | Filter bubbles / cocoons; Exposure diversity | Addresses how recommendation design interacts with exposure diversity, filter bubbles, or polarization-related outcomes. | User-controllable Recommendation Against Filter Bubbles |
| Areeb, QM (2023) | Social media | Social-media recommendation and information exposure | Review / bibliometric | Published literature/document corpus; number of records not captured in the supplied coding sheet. | Filter bubbles / cocoons; Echo chambers; Exposure diversity | Synthesizes prior work and maps major themes, measures, and gaps around recommender-driven bubbles, chambers, or polarization. | Filter bubbles in recommender systems: Fact or fallacy-A systematic review |
| De Biasio, A (2023) | Social network | Online social-network recommendation and network exposure | System / algorithm design | System/algorithm evaluation data; specific benchmark/sample size not captured in the supplied coding sheet. | Echo chambers; Exposure diversity | Emphasizes diversity- or serendipity-oriented design as a way to counter homogenization in recommendations. | On the problem of recommendation for sensitive users and influential items: Simultaneously maintaining interest and diversity |
| Du, Y.R. (2023) | News app | AI-powered/news-app recommendation and news exposure | Qualitative study | Qualitative evidence; participant/document sample size not captured in the supplied coding sheet. | Echo chambers; Exposure diversity | Addresses how recommendation design interacts with exposure diversity, filter bubbles, or polarization-related outcomes. | Personalization, Echo Chambers, News Literacy, and Algorithmic Literacy: A Qualitative Study of AI-Powered News App Users |
| Knudsen, E. (2023) | News / news recommender | News recommendation and online news exposure | Experiment | Experimental evidence; participant/dataset size not captured in the supplied coding sheet. | Filter bubbles / cocoons; Selective exposure | Shows recommender effects on selective exposure vary by users’ prior ideology and conditions of use. | Modeling news recommender systems’ conditional effects on selective exposure: evidence from two online experiments |
| Lieto, A. (2023) | Art recommender | Art recommendation, diversity, and social inclusion | Modeling / simulation | Model/simulation-based evidence; specific dataset/sample size not captured in the supplied coding sheet. | Filter bubbles / cocoons; Exposure diversity | Emphasizes diversity- or serendipity-oriented design as a way to counter homogenization in recommendations. | DEGARI 2.0: A diversity-seeking, explainable, and affective art recommender for social inclusion |
| Ludwig, K. (2023) | News / news recommender | News recommendation and online news exposure | Experiment | Experimental evidence; participant/dataset size not captured in the supplied coding sheet. | Polarization; Filter bubbles / cocoons | Shows news recommendation systems materially affect viewpoint diversity, topic diversity, or polarization-related exposure patterns. | Divided by the Algorithm? The (Limited) Effects of Content- and Sentiment-Based News Recommendation on Affective, Ideological, and Perceived Polarization |
| Makhortykh, M. (2023) | News / news recommender | News recommendation and online news exposure | System / algorithm design | System/algorithm evaluation data; specific benchmark/sample size not captured in the supplied coding sheet. | Filter bubbles / cocoons | Shows news recommendation systems materially affect viewpoint diversity, topic diversity, or polarization-related exposure patterns. | Can Filter Bubbles Protect Information Freedom? Discussions of Algorithmic News Recommenders in Eastern Europe |
| Michiels, L (2023) | News / news recommender | News recommendation and online news exposure | Modeling / simulation | Model/simulation-based evidence; specific dataset/sample size not captured in the supplied coding sheet. | Filter bubbles / cocoons; Exposure diversity | Proposes a measurement framework for quantifying filter-bubble effects rather than assuming them. | How ShouldWe Measure Filter Bubbles? A Regression Model and Evidence for Online News |
| Pathak, R (2023) | Twitter/X | Twitter/X recommendation, exposure, and network dynamics | Modeling / simulation | Model/simulation-based evidence; specific dataset/sample size not captured in the supplied coding sheet. | Filter bubbles / cocoons; Misinformation / radicalization | Examines how recommendation logic can amplify misinformation spread and how interventions may reduce that risk. | Understanding the Contribution of Recommendation Algorithms on Misinformation Recommendation and Misinformation Dissemination on Social Networks |
| Zhang, H (2023) | News / news recommender | News recommendation and online news exposure | Experiment | Experimental evidence; participant/dataset size not captured in the supplied coding sheet. | Polarization; Filter bubbles / cocoons | Shows news recommendation systems materially affect viewpoint diversity, topic diversity, or polarization-related exposure patterns. | Evolution of Filter Bubbles and Polarization in News Recommendation |
| Duskin, K (2024) | Twitter/X | Twitter/X recommendation, exposure, and network dynamics | Experiment | Experimental evidence; participant/dataset size not captured in the supplied coding sheet. | Echo chambers; Misinformation / radicalization | Audits recommendation outputs to detect whether the system steers users toward ideological clustering, misinformation, or echo chambers. | Echo Chambers in the Age of Algorithms: An Audit of Twitter's Friend Recommender System |
| Gao, CM (2024) | General recommender systems | General recommender-system design/evaluation | Experiment | Experimental evidence; participant/dataset size not captured in the supplied coding sheet. | Filter bubbles / cocoons | Designs or tests diversification strategies intended to reduce homogeneity while preserving recommendation relevance. | CIRS: Bursting Filter Bubbles by Counterfactual Interactive Recommender System |
| Jeng, JH (2024) | News / news recommender | News recommendation and online news exposure | Measurement / empirical modeling | Empirical/measurement data; specific dataset/sample size not captured in the supplied coding sheet. | Polarization; Selective exposure; Exposure diversity | Designs or tests diversification strategies intended to reduce homogeneity while preserving recommendation relevance. | Bridging Viewpoints in News with Recommender Systems |
| Jiang, T. (2024) | News / news recommender | News recommendation and online news exposure | Measurement / empirical modeling | Empirical/measurement data; specific dataset/sample size not captured in the supplied coding sheet. | Filter bubbles / cocoons | Addresses how recommendation design interacts with exposure diversity, filter bubbles, or polarization-related outcomes. | Breaking the Filter Bubbles: The Effect of Algorithm Curation on Selectivity of Information Consumption and Attitude Extremity; 应对过滤气泡: 算法策展对用户信息消费行为选择性和态度极端化的影响 |
| Lechiakh, M. (2024) | General recommender systems | General recommender-system design/evaluation | Experiment | Experimental evidence; participant/dataset size not captured in the supplied coding sheet. | Polarization; Filter bubbles / cocoons | Designs or tests diversification strategies intended to reduce homogeneity while preserving recommendation relevance. | Towards long-term depolarized interactive recommendations |
| Nechushtai, E. (2024) | YouTube | YouTube recommendation and content exposure | Audit | Platform/audit observations; specific audit sample size not captured in the supplied coding sheet. | Polarization; Filter bubbles / cocoons | Examines how personalization on YouTube shapes exposure diversity and can channel users toward conspiratorial or homogeneous content. | More of the Same? Homogenization in News Recommendations When Users Search on Google, YouTube, Facebook, and Twitter |
| Pathak, R (2024) | Twitter/X | Twitter/X recommendation, exposure, and network dynamics | Modeling / simulation | Model/simulation-based evidence; specific dataset/sample size not captured in the supplied coding sheet. | Filter bubbles / cocoons; Echo chambers; Exposure diversity | Examines how recommendation logic can amplify misinformation spread and how interventions may reduce that risk. | Analyzing the Interplay between Diversity of News Recommendations and Misinformation Spread in Social Media |
| Pathak, R. (2024) | Twitter/X | Twitter/X recommendation, exposure, and network dynamics | System / algorithm design | System/algorithm evaluation data; specific benchmark/sample size not captured in the supplied coding sheet. | Filter bubbles / cocoons; Echo chambers; Misinformation / radicalization | Examines how recommendation logic can amplify misinformation spread and how interventions may reduce that risk. | Advancing Misinformation Awareness in Recommender Systems for Social Media Information Integrity |
| Reid, J. (2024) | General recommender systems | General recommender-system design/evaluation | Modeling / simulation | Model/simulation-based evidence; specific dataset/sample size not captured in the supplied coding sheet. | Filter bubbles / cocoons; Exposure diversity | Emphasizes diversity- or serendipity-oriented design as a way to counter homogenization in recommendations. | Digitising “The Big Lie”: Algorithmic Curation as an Inhibitor of Media Exposure Diversity Online |
| Sergas, U (2024) | News / news recommender | News recommendation and online news exposure | Modeling / simulation | Model/simulation-based evidence; specific dataset/sample size not captured in the supplied coding sheet. | Polarization; Filter bubbles / cocoons; Echo chambers | Shows news recommendation systems materially affect viewpoint diversity, topic diversity, or polarization-related exposure patterns. | Perspective Diversification News Recommender System |
| Shin, D. (2024) | TikTok | TikTok recommendation and content exposure | Audit | Platform/audit observations; specific audit sample size not captured in the supplied coding sheet. | Polarization; Filter bubbles / cocoons; Misinformation / radicalization | Audits recommendation outputs to detect whether the system steers users toward ideological clustering, misinformation, or echo chambers. | How Algorithms Promote Self-Radicalization: Audit of TikTok’s Algorithm Using a Reverse Engineering Method |
| Sonoda, A (2024) | News / news recommender | News recommendation and online news exposure | Experiment | Experimental evidence; participant/dataset size not captured in the supplied coding sheet. | Echo chambers; Exposure diversity | Emphasizes diversity- or serendipity-oriented design as a way to counter homogenization in recommendations. | User Experiments on the Effect of the Diversity of Consumption on News Services |
| Treuillier, C (2024) | News / news recommender | News recommendation and online news exposure | Experiment | Experimental evidence; participant/dataset size not captured in the supplied coding sheet. | Filter bubbles / cocoons; Exposure diversity | Shows news recommendation systems materially affect viewpoint diversity, topic diversity, or polarization-related exposure patterns. | Beyond Trade-offs: Unveiling Fairness-Constrained Diversity in News Recommender Systems |
| Xu, YB (2024) | General recommender systems | General recommender-system design/evaluation | Experiment | Experimental evidence; participant/dataset size not captured in the supplied coding sheet. | Filter bubbles / cocoons; Exposure diversity | Designs or tests diversification strategies intended to reduce homogeneity while preserving recommendation relevance. | GS2-RS: A Generative Approach for Alleviating ColdStart and Filter Bubbles in Recommender Systems |
| Zhou, X. (2024) | General recommender systems | General recommender-system design/evaluation | System / algorithm design | System/algorithm evaluation data; specific benchmark/sample size not captured in the supplied coding sheet. | Filter bubbles / cocoons; Exposure diversity | Emphasizes diversity- or serendipity-oriented design as a way to counter homogenization in recommendations. | "Bee and I need diversity!" Break Filter Bubbles in Recommendation Systems through Embodied AI Learning |
| Ahmmad, M. (2025) | YouTube | YouTube recommendation and content exposure | Review / bibliometric | Published literature/document corpus; number of records not captured in the supplied coding sheet. | Polarization; Filter bubbles / cocoons; Echo chambers | Synthesizes prior work and maps major themes, measures, and gaps around recommender-driven bubbles, chambers, or polarization. | Trap of Social Media Algorithms: A Systematic Review of Research on Filter Bubbles, Echo Chambers, and Their Impact on Youth |
| Alam, MJ (2025) | Social network | Online social-network recommendation and network exposure | Experiment | Experimental evidence; participant/dataset size not captured in the supplied coding sheet. | Polarization; Echo chambers; Exposure diversity | Models echo-chamber dynamics and evaluates how recommendation or network interventions alter polarization trajectories. | Combating Echo Chambers in Online Social Network by Increasing Content Diversity in Recommendation |
| Bojic, L (2025) | Social media | Social-media recommendation and information exposure | Qualitative study | Qualitative evidence; participant/document sample size not captured in the supplied coding sheet. | Polarization; Exposure diversity | Uses simulation to show how repeated personalized exposure can generate or intensify polarization over time. | An Agent-Based Simulation of Politicized Topics Using Large Language Models: Algorithmic Personalization and Polarization on Social Media |
| Han, G (2025) | General recommender systems | General recommender-system design/evaluation | Experiment | Experimental evidence; participant/dataset size not captured in the supplied coding sheet. | Filter bubbles / cocoons; Exposure diversity | Emphasizes diversity- or serendipity-oriented design as a way to counter homogenization in recommendations. | Controlling Diversity at Inference: Guiding Diffusion Recommender Models with Targeted Category Preferences |
| Jiang, T. (2025) | General recommender systems | General recommender-system design/evaluation | Experiment | Experimental evidence; participant/dataset size not captured in the supplied coding sheet. | Filter bubbles / cocoons; Exposure diversity | Addresses how recommendation design interacts with exposure diversity, filter bubbles, or polarization-related outcomes. | Restraining the formation of filter bubbles with algorithmic affordances: Toward more balanced information consumption and decreased attitude extremity |
| Kim, S (2025) | General recommender systems | General recommender-system design/evaluation | Experiment | Experimental evidence; participant/dataset size not captured in the supplied coding sheet. | Filter bubbles / cocoons; Exposure diversity | Emphasizes diversity- or serendipity-oriented design as a way to counter homogenization in recommendations. | Balancing Diversity in Session-Based Recommendation Between Relevance and Unexpectedness |
| Ludwig, K. (2025) | News / news recommender | News recommendation and online news exposure | Experiment | Experimental evidence; participant/dataset size not captured in the supplied coding sheet. | Polarization; Filter bubbles / cocoons | Experimental evidence shows ideology-based news recommendation can polarize, but effects are bounded and context dependent. | Putting ‘filter bubble’ effects to the test: evidence on the polarizing impact of ideology-based news recommendation from two experiments in Germany and the U.S. |
| Pathak, R (2025) | News / news recommender | News recommendation and online news exposure | Modeling / simulation | Model/simulation-based evidence; specific dataset/sample size not captured in the supplied coding sheet. | Filter bubbles / cocoons; Misinformation / radicalization | Examines how recommendation logic can amplify misinformation spread and how interventions may reduce that risk. | Circumventing Misinformation Controls: Assessing the Robustness of Intervention Strategies in Recommender Systems |
| Poechhacker, N. (2025) | General recommender systems | General recommender-system design/evaluation | System / algorithm design | System/algorithm evaluation data; specific benchmark/sample size not captured in the supplied coding sheet. | Filter bubbles / cocoons | Addresses how recommendation design interacts with exposure diversity, filter bubbles, or polarization-related outcomes. | Recommender Systems beyond the Filter Bubble: Algorithmic Media and the Fabrication of Publics |
| Ren, BF (2025) | General recommender systems | General recommender-system design/evaluation | Experiment | Experimental evidence; participant/dataset size not captured in the supplied coding sheet. | Filter bubbles / cocoons; Exposure diversity | Emphasizes diversity- or serendipity-oriented design as a way to counter homogenization in recommendations. | Leveraging personalized diversity level for recommendations with knowledge graph |
| Schellingerhout, R. (2025) | YouTube | YouTube recommendation and content exposure | Experiment | Experimental evidence; participant/dataset size not captured in the supplied coding sheet. | Filter bubbles / cocoons; Misinformation / radicalization | Examines how personalization on YouTube shapes exposure diversity and can channel users toward conspiratorial or homogeneous content. | Accounting for Personalization in Personalization Algorithms: YouTube’s Treatment of Conspiracy Content |
| Wang, R. (2025) | News / news recommender | News recommendation and online news exposure | Modeling / simulation | Model/simulation-based evidence; specific dataset/sample size not captured in the supplied coding sheet. | Filter bubbles / cocoons; Selective exposure; Exposure diversity | Shows news recommendation systems materially affect viewpoint diversity, topic diversity, or polarization-related exposure patterns. | How News Recommendation System Effects User’s Individual and Aggregate Topic Diversity—A Study of Simulation Analysis |
| Xi, YJ (2025) | General recommender systems | General recommender-system design/evaluation | Experiment | Experimental evidence; participant/dataset size not captured in the supplied coding sheet. | Filter bubbles / cocoons; Exposure diversity | Designs or tests diversification strategies intended to reduce homogeneity while preserving recommendation relevance. | Bursting Filter Bubble: Enhancing Serendipity Recommendations with Aligned Large Language Models |
| Yan, C. (2025) | Twitter/X | Twitter/X recommendation, exposure, and network dynamics | Review / bibliometric | Published literature/document corpus; number of records not captured in the supplied coding sheet. | Polarization; Filter bubbles / cocoons; Echo chambers | Synthesizes prior work and maps major themes, measures, and gaps around recommender-driven bubbles, chambers, or polarization. | Knowledge mapping of information cocoons: A bibliometric study using visual analysis |
| Yu, DN (2025) | Social network | Online social-network recommendation and network exposure | Experiment | Experimental evidence; participant/dataset size not captured in the supplied coding sheet. | Echo chambers; Exposure diversity | Addresses how recommendation design interacts with exposure diversity, filter bubbles, or polarization-related outcomes. | Breaking the Loop: Causal Learning to Mitigate Echo Chambers in Social Networks |
| Zhang, MY (2025) | News / news recommender | News recommendation and online news exposure | Experiment | Experimental evidence; participant/dataset size not captured in the supplied coding sheet. | Polarization; Misinformation / radicalization | Examines how recommendation logic can amplify misinformation spread and how interventions may reduce that risk. | How Misinformation Diffuses on Online Social Networks: Radical Opinions, Adaptive Relationship, and Algorithmic Intervention |
| Zheng, Y. (2025) | Conversational recommender | Conversational recommendation and preference learning | Modeling / simulation | Model/simulation-based evidence; specific dataset/sample size not captured in the supplied coding sheet. | Filter bubbles / cocoons | Designs or tests diversification strategies intended to reduce homogeneity while preserving recommendation relevance. | HyperCRS: Hypergraph-Aware Multi-Grained Preference Learning to Burst Filter Bubbles in Conversational Recommendation System |
