# Supplementary material for: Consensus or conflict? Recommender systems as recursive exposure systems in the production of polarization
Source: Front Sociol. 2026 Sep 30;11:1940431. doi: 10.3389/fsoc.2026.1940431 (PMC13662418; doi:10.3389/fsoc.2026.1940431)
Supplement: Supplementary file 4 [file Table_4.DOCX]

**Supplementary Table S4. RQ-Linked Evidentiary Map of the 60 Included Studies**

This table maps each included study to the research question(s) to which it contributes, the study-specific evidence represented in the systematic coding, and the role of that evidence in the cross-study synthesis. RQ assignment is a review-level analytical mapping and does not imply that the original study was designed to answer the review's RQs.

| **Study** | **RQ(s)** | **Extracted evidence** | **Contribution to synthesis / conclusion** |
| --- | --- | --- | --- |
| Aridor G (2020) | RQ2, RQ3 | Links recommendation design to exposure diversity/bubble-related outcomes. | Contributes computational evidence on how the focal mechanism is operationalized; interpreted primarily as mechanism plausibility. Supplies component-level evidence used in PMPF integration; it does not establish the complete recursive pathway. |
| Joris G. (2020) | RQ1, RQ3 | Establishes an interdisciplinary framework for assessing news diversity and recommendation systems; does not directly test recommender effects. | Informs the conceptualization and boundary-setting of filter-bubble/exposure phenomena. Contributes conceptual/theoretical feedback logic to PMPF rather than direct demonstration of recursion. |
| Lunardi GM (2020) | RQ2, RQ3 | Proposes/uses measurement of filter-bubble effects. | Contributes computational evidence on how the focal mechanism is operationalized; interpreted primarily as mechanism plausibility. Supplies component-level evidence used in PMPF integration; it does not establish the complete recursive pathway. |
| Sun J. (2020) | RQ2, RQ3 | Evaluates cross-domain recommendation as a strategy to alleviate filter bubbles while preserving recommendation relevance. | Contributes experimental/intervention evidence on conditional recommender, exposure, or behavioural effects. Contributes conceptual/theoretical feedback logic to PMPF rather than direct demonstration of recursion. |
| Valdez AC (2020) | RQ2, RQ3 | Examines how human behaviour and algorithmic processes jointly contribute to online misinformation and its amplification. | Contributes computational evidence on how the focal mechanism is operationalized; interpreted primarily as mechanism plausibility. Contributes conceptual/theoretical feedback logic to PMPF rather than direct demonstration of recursion. |
| Vrijenhoek S (2020) | RQ1, RQ2, RQ3 | Proposes a framework and diversity metrics for assessing normative diversity in news recommendations; reports an ongoing project rather than completed effects. | Informs the conceptualization and boundary-setting of filter-bubble/exposure phenomena. Contributes evidence on the operational examination of the focal construct. Contributes conceptual/theoretical feedback logic to PMPF rather than direct demonstration of recursion. |
| Donkers T (2021) | RQ2, RQ3 | Models echo-chamber dynamics and polarization trajectories. | Contributes computational evidence on how the focal mechanism is operationalized; interpreted primarily as mechanism plausibility. Supports the integrative synthesis with direct evidence of a recursive return pathway. |
| Grossetti Q (2021) | RQ2, RQ3 | Finds network ranking can reinforce homophily; diversity can counter it. | Contributes design-based evidence on how recommender interventions alter exposure or relational conditions. Supplies component-level evidence used in PMPF integration; it does not establish the complete recursive pathway. |
| Morales PR (2021) | RQ2, RQ3 | Audits how social-network recommendations can alter ideological structure and polarization in modeled ideological spaces. | Contributes observational/audit evidence on recommender outputs or exposure patterns. Supports the integrative synthesis with direct evidence of a recursive return pathway. |
| Tommasel A (2021) | RQ2, RQ3 | Develops friend recommendations that connect users outside their echo chambers while accounting for user interests. | Contributes design-based evidence on how recommender interventions alter exposure or relational conditions. Supplies component-level evidence used in PMPF integration; it does not establish the complete recursive pathway. |
| Vrijenhoek S (2021) | RQ2, RQ3 | Finds changes in viewpoint/topic diversity or polarization-related exposure. | Contributes computational evidence on how the focal mechanism is operationalized; interpreted primarily as mechanism plausibility. Contributes conceptual/theoretical feedback logic to PMPF rather than direct demonstration of recursion. |
| Zhao X (2021) | RQ2, RQ3 | Links recommendation design to exposure diversity/bubble-related outcomes. | Contributes design-based evidence on how recommender interventions alter exposure or relational conditions. Supplies component-level evidence used in PMPF integration; it does not establish the complete recursive pathway. |
| El-Moutaouakkil Z (2022) | RQ2, RQ3 | Models the trade-off between recommendation accuracy and limiting polarization in personalized recommendation. | Contributes computational evidence on how the focal mechanism is operationalized; interpreted primarily as mechanism plausibility. Contributes to cross-level integration without demonstrating recursive feedback directly. |
| Gao ZL (2022) | RQ2 | Tests targeted diversification to mitigate filter bubbles while maintaining recommendation relevance. | Contributes experimental/intervention evidence on conditional recommender, exposure, or behavioural effects. |
| Han H (2022) | RQ2, RQ3 | Evaluates filter-bubble evolution in personalized news recommendation, showing convergence between user interests and recommended content over repeated interactions. | Contributes observational/audit evidence on recommender outputs or exposure patterns. Supports the integrative synthesis with direct evidence of a recursive return pathway. |
| Sonoda A. (2022) | RQ2, RQ3 | Supports diversity/serendipity design against homogenization. | Contributes experimental/intervention evidence on conditional recommender, exposure, or behavioural effects. Contributes to cross-level integration without demonstrating recursive feedback directly. |
| Stray J. (2022) | RQ1, RQ2, RQ3 | Synthesizes design approaches for recommender systems intended to reduce polarization and identifies relevant intervention objectives. | Informs the distinction between polarization outcomes and exposure-level phenomena. Contributes evidence on the operational examination of the focal construct. Contributes conceptual/theoretical feedback logic to PMPF rather than direct demonstration of recursion. |
| Wang WJ (2022) | RQ2, RQ3 | Links recommendation design to exposure diversity/bubble-related outcomes. | Contributes experimental/intervention evidence on conditional recommender, exposure, or behavioural effects. Supports the integrative synthesis with direct evidence of a recursive return pathway. |
| Areeb QM (2023) | RQ1, RQ3 | Maps themes, measures, and gaps in bubbles/chambers/polarization research. | Informs the conceptualization and boundary-setting of filter-bubble/exposure phenomena. Contributes conceptual/theoretical feedback logic to PMPF rather than direct demonstration of recursion. |
| De Biasio A (2023) | RQ2, RQ3 | Supports diversity/serendipity design against homogenization. | Contributes design-based evidence on how recommender interventions alter exposure or relational conditions. Supplies component-level evidence used in PMPF integration; it does not establish the complete recursive pathway. |
| Du Y.R. (2023) | RQ1, RQ2, RQ3 | Shows how users interpret personalization and echo chambers in AI-powered news apps, highlighting the roles of news and algorithmic literacy. | Informs the conceptual treatment of echo chambers as relational/network configurations. Contributes interpretive evidence on users' understandings and experiences of recommender-mediated processes. Supplies component-level evidence used in PMPF integration; it does not establish the complete recursive pathway. |
| Knudsen E. (2023) | RQ2, RQ3 | Selective-exposure effects vary by prior ideology and use conditions. | Contributes experimental/intervention evidence on conditional recommender, exposure, or behavioural effects. Contributes to cross-level integration without demonstrating recursive feedback directly. |
| Lieto A. (2023) | RQ2 | Supports diversity/serendipity design against homogenization. | Contributes computational evidence on how the focal mechanism is operationalized; interpreted primarily as mechanism plausibility. |
| Ludwig K. (2023) | RQ2, RQ3 | Finds limited and conditional effects of news recommendation on affective, ideological, and perceived polarization. | Contributes experimental/intervention evidence on conditional recommender, exposure, or behavioural effects. Contributes to cross-level integration without demonstrating recursive feedback directly. |
| Makhortykh M. (2023) | RQ1, RQ3 | Examines how algorithmic news recommenders and filter bubbles are discussed in Eastern European information-freedom debates. | Informs the conceptualization and boundary-setting of filter-bubble/exposure phenomena. Contributes conceptual/theoretical feedback logic to PMPF rather than direct demonstration of recursion. |
| Michiels L (2023) | RQ2, RQ3 | Proposes/uses measurement of filter-bubble effects. | Contributes computational evidence on how the focal mechanism is operationalized; interpreted primarily as mechanism plausibility. Supplies component-level evidence used in PMPF integration; it does not establish the complete recursive pathway. |
| Pathak R (2023) | RQ2, RQ3 | Examines misinformation amplification and possible mitigation. | Contributes computational evidence on how the focal mechanism is operationalized; interpreted primarily as mechanism plausibility. Supplies component-level evidence used in PMPF integration; it does not establish the complete recursive pathway. |
| Zhang H (2023) | RQ2, RQ3 | Simulates the long-run evolution of filter bubbles and polarization under news recommendation. | Contributes computational evidence on how the focal mechanism is operationalized; interpreted primarily as mechanism plausibility. Supports the integrative synthesis with direct evidence of a recursive return pathway. |
| Duskin K (2024) | RQ2, RQ3 | Audits Twitter's friend recommender and finds recommendation patterns capable of increasing network homogeneity and echo-chamber formation. | Contributes observational/audit evidence on recommender outputs or exposure patterns. Supports the integrative synthesis with direct evidence of a recursive return pathway. |
| Gao CM (2024) | RQ2, RQ3 | Tests diversification intended to reduce homogeneity while retaining relevance. | Contributes experimental/intervention evidence on conditional recommender, exposure, or behavioural effects. Supports the integrative synthesis with direct evidence of a recursive return pathway. |
| Jeng JH (2024) | RQ3 | Proposes and motivates viewpoint-bridging news recommender interventions; the contribution describes a research program rather than completed polarization effects. | Contributes to cross-level integration without demonstrating recursive feedback directly. |
| Jiang T. (2024) | RQ2, RQ3 | Examines how algorithmic curation affects selective information consumption and attitude extremity. | Contributes computational evidence on how the focal mechanism is operationalized; interpreted primarily as mechanism plausibility. Contributes to cross-level integration without demonstrating recursive feedback directly. |
| Lechiakh M. (2024) | RQ2, RQ3 | Tests diversification intended to reduce homogeneity while retaining relevance. | Contributes experimental/intervention evidence on conditional recommender, exposure, or behavioural effects. Supports the integrative synthesis with direct evidence of a recursive return pathway. |
| Nechushtai E. (2024) | RQ2 | Finds substantial homogenization in top-ranked news recommendations across major search and social platforms. | Contributes observational/audit evidence on recommender outputs or exposure patterns. |
| Pathak R (2024) | RQ2, RQ3 | Examines misinformation amplification and possible mitigation. | Contributes computational evidence on how the focal mechanism is operationalized; interpreted primarily as mechanism plausibility. Contributes to cross-level integration without demonstrating recursive feedback directly. |
| Pathak R. (2024) | RQ2, RQ3 | Examines misinformation amplification and possible mitigation. | Contributes design-based evidence on how recommender interventions alter exposure or relational conditions. Contributes conceptual/theoretical feedback logic to PMPF rather than direct demonstration of recursion. |
| Reid J. (2024) | RQ1, RQ3 | Argues that algorithmic curation can constrain media-exposure diversity and thereby contribute to filter-bubble conditions. | Informs the conceptualization and boundary-setting of filter-bubble/exposure phenomena. Contributes conceptual/theoretical feedback logic to PMPF rather than direct demonstration of recursion. |
| Sergas U (2024) | RQ2, RQ3 | Develops a perspective-diversification news recommender intended to broaden viewpoint exposure; downstream polarization is not directly tested. | Contributes design-based evidence on how recommender interventions alter exposure or relational conditions. Contributes to cross-level integration without demonstrating recursive feedback directly. |
| Shin D. (2024) | RQ2, RQ3 | Audit detects clustering, misinformation, or echo-chamber steering. | Contributes observational/audit evidence on recommender outputs or exposure patterns. Supports the integrative synthesis with direct evidence of a recursive return pathway. |
| Sonoda A (2024) | RQ2, RQ3 | Supports diversity/serendipity design against homogenization. | Contributes experimental/intervention evidence on conditional recommender, exposure, or behavioural effects. Contributes to cross-level integration without demonstrating recursive feedback directly. |
| Treuillier C (2024) | RQ2 | Evaluates fairness-constrained diversification in news recommendation, examining trade-offs among relevance, diversity, and fairness. | Contributes experimental/intervention evidence on conditional recommender, exposure, or behavioural effects. |
| Xu YB (2024) | RQ2 | Tests diversification intended to reduce homogeneity while retaining relevance. | Contributes experimental/intervention evidence on conditional recommender, exposure, or behavioural effects. |
| Zhou X. (2024) | RQ2 | Evaluates an embodied AI-literacy intervention designed to help users understand how repeated choices can contribute to filter bubbles. | Contributes experimental/intervention evidence on conditional recommender, exposure, or behavioural effects. |
| Ahmmad M. (2025) | RQ1, RQ3 | Maps themes, measures, and gaps in bubbles/chambers/polarization research. | Informs the distinction between polarization outcomes and exposure-level phenomena. Contributes conceptual/theoretical feedback logic to PMPF rather than direct demonstration of recursion. |
| Alam MJ (2025) | RQ2, RQ3 | Evaluates whether increasing recommendation diversity can mitigate echo-chamber formation in a simulated social-network setting. | Contributes experimental/intervention evidence on conditional recommender, exposure, or behavioural effects. Contributes to cross-level integration without demonstrating recursive feedback directly. |
| Bojic L (2025) | RQ2, RQ3 | Uses an LLM-based agent simulation in which personalized exposure and user responses recursively shape subsequent content, producing polarization under specified conditions. | Contributes computational evidence on how the focal mechanism is operationalized; interpreted primarily as mechanism plausibility. Supports the integrative synthesis with direct evidence of a recursive return pathway. |
| Han G (2025) | RQ2 | Supports diversity/serendipity design against homogenization. | Contributes experimental/intervention evidence on conditional recommender, exposure, or behavioural effects. |
| Jiang T. (2025) | RQ2 | Links recommendation design to exposure diversity/bubble-related outcomes. | Contributes experimental/intervention evidence on conditional recommender, exposure, or behavioural effects. |
| Kim S (2025) | RQ2 | Supports diversity/serendipity design against homogenization. | Contributes experimental/intervention evidence on conditional recommender, exposure, or behavioural effects. |
| Ludwig K. (2025) | RQ2, RQ3 | Ideology-based news recommendation shows bounded, context-dependent polarization. | Contributes experimental/intervention evidence on conditional recommender, exposure, or behavioural effects. Contributes to cross-level integration without demonstrating recursive feedback directly. |
| Pathak R (2025) | RQ2, RQ3 | Examines misinformation amplification and possible mitigation. | Contributes computational evidence on how the focal mechanism is operationalized; interpreted primarily as mechanism plausibility. Contributes to cross-level integration without demonstrating recursive feedback directly. |
| Poechhacker N. (2025) | RQ2, RQ3 | Links recommendation design to exposure diversity/bubble-related outcomes. | Contributes design-based evidence on how recommender interventions alter exposure or relational conditions. Contributes conceptual/theoretical feedback logic to PMPF rather than direct demonstration of recursion. |
| Ren BF (2025) | RQ2, RQ3 | Supports diversity/serendipity design against homogenization. | Contributes experimental/intervention evidence on conditional recommender, exposure, or behavioural effects. Supplies component-level evidence used in PMPF integration; it does not establish the complete recursive pathway. |
| Schellingerhout R. (2025) | RQ2, RQ3 | YouTube personalization shapes diversity and may channel homogeneous/conspiratorial exposure. | Contributes experimental/intervention evidence on conditional recommender, exposure, or behavioural effects. Supports the integrative synthesis with direct evidence of a recursive return pathway. |
| Wang R. (2025) | RQ2, RQ3 | Finds changes in viewpoint/topic diversity or polarization-related exposure. | Contributes computational evidence on how the focal mechanism is operationalized; interpreted primarily as mechanism plausibility. Supports the integrative synthesis with direct evidence of a recursive return pathway. |
| Xi YJ (2025) | RQ2, RQ3 | Tests diversification intended to reduce homogeneity while retaining relevance. | Contributes experimental/intervention evidence on conditional recommender, exposure, or behavioural effects. Contributes conceptual/theoretical feedback logic to PMPF rather than direct demonstration of recursion. |
| Yan C. (2025) | RQ1, RQ3 | Maps the intellectual structure, themes, and development of information-cocoon research through bibliometric analysis. | Informs the conceptualization and boundary-setting of filter-bubble/exposure phenomena. Contributes conceptual/theoretical feedback logic to PMPF rather than direct demonstration of recursion. |
| Yu DN (2025) | RQ2, RQ3 | Links recommendation design to exposure diversity/bubble-related outcomes. | Contributes experimental/intervention evidence on conditional recommender, exposure, or behavioural effects. Supplies component-level evidence used in PMPF integration; it does not establish the complete recursive pathway. |
| Zhang MY (2025) | RQ2, RQ3 | Examines misinformation amplification and possible mitigation. | Contributes experimental/intervention evidence on conditional recommender, exposure, or behavioural effects. Supports the integrative synthesis with direct evidence of a recursive return pathway. |
| Zheng Y. (2025) | RQ2, RQ3 | Develops and benchmarks a hypergraph-aware conversational recommender designed to improve preference diversity and mitigate filter-bubble effects. | Contributes design-based evidence on how recommender interventions alter exposure or relational conditions. Supports the integrative synthesis with direct evidence of a recursive return pathway. |

**Notes.** RQ1 = How are filter bubbles, echo chambers, and polarization conceptualized in the literature on recommender systems? RQ2 = How are these phenomena operationalized and examined across different methodological approaches? RQ3 = How can evidence across algorithmic, behavioural, relational, and polarization outcomes be integrated within a recursive framework of platform-mediated communication? Studies may contribute to more than one RQ. RQ3 assignment indicates contribution to the integrative synthesis and does not by itself indicate direct recursive evidence. Direct, partial/component, conceptual/theoretical, and no-recursivity classifications remain separately reported in Supplementary Table S2.
